# Plastic additive components of PM_2.5_ increase corrected QT interval: Screening for exposure markers based on airborne exposome

**DOI:** 10.1093/pnasnexus/pgad397

**Published:** 2023-11-23

**Authors:** Xiaotu Liu, Yanwen Wang, Jianlong Fang, Renjie Chen, Yue Sun, Shuqin Tang, Minghao Wang, Haidong Kan, Tiantian Li, Da Chen

**Affiliations:** School of Environment and Guangdong Key Laboratory of Environmental Pollution and Health, Jinan University, Guangzhou 511443, China; China CDC Key Laboratory of Environment and Population Health, National Institute of Environmental Health, Chinese Center for Disease Control and Prevention, Beijing 100021, China; China CDC Key Laboratory of Environment and Population Health, National Institute of Environmental Health, Chinese Center for Disease Control and Prevention, Beijing 100021, China; School of Public Health, Key Lab of Public Health Safety of the Ministry of Education and NHC Key Lab of Health Technology Assessment, Shanghai Institute of Infectious Disease and Biosecurity, Fudan University, Shanghai 200032, China; China CDC Key Laboratory of Environment and Population Health, National Institute of Environmental Health, Chinese Center for Disease Control and Prevention, Beijing 100021, China; School of Environment and Guangdong Key Laboratory of Environmental Pollution and Health, Jinan University, Guangzhou 511443, China; China CDC Key Laboratory of Environment and Population Health, National Institute of Environmental Health, Chinese Center for Disease Control and Prevention, Beijing 100021, China; School of Public Health, Key Lab of Public Health Safety of the Ministry of Education and NHC Key Lab of Health Technology Assessment, Shanghai Institute of Infectious Disease and Biosecurity, Fudan University, Shanghai 200032, China; China CDC Key Laboratory of Environment and Population Health, National Institute of Environmental Health, Chinese Center for Disease Control and Prevention, Beijing 100021, China; School of Environment and Guangdong Key Laboratory of Environmental Pollution and Health, Jinan University, Guangzhou 511443, China

**Keywords:** ambient fine particles, plastic additives, QT_C_ interval, exposure markers

## Abstract

The impact of industrial chemical components of ambient fine particles (e.g. PM_2.5_) on cardiovascular health has been poorly explored. Our study reports for the first time the associations between human exposure to complex plastic additive (PA) components of PM_2.5_ and prolongation of heart rate–corrected QT (QT_C_) interval by employing a screening-to-validation strategy based on a cohort of 373 participants (136 in the screening set and 237 in the validation set) recruited from 7 communities across China. The high-throughput airborne exposome framework revealed ubiquitous occurrences of 95 of 224 target PAs in PM_2.5_, totaling from 66.3 to 555 ng m^−3^ across the study locations. Joint effects were identified for 9 of the 13 groups of PAs with positive associations with QT_C_ interval. Independent effect analysis also identified and validated tris(2-chloroisopropyl) phosphate, di-n-butyl/diisobutyl adipate, and 3,5-di-tert-butyl-4-hydroxybenzaldehyde as the key exposure markers for QT_C_ interval prolongation and changes of selected cardiovascular biomarkers. Our findings highlight the important contributions of airborne industrial chemicals to the risks of cardiovascular diseases and underline the critical need for further research on the underlying mechanisms, toxic modes of action, and human exposure risks.

**Significance Statement**

Air pollution constitutes an important risk factor in the etiology of cardiovascular diseases. While exposure to ambient fine particles (e.g. PM_2.5_) has been well reported to impact cardiovascular health, the role of industrial chemical components of PM_2.5_ in health effects has been poorly evaluated. Our work reveals the occurrences of a complexity of plastic additive chemicals in airborne fine particles, demonstrating ubiquitous human exposure risks. The screening-to-validation strategy demonstrates that human exposure to complex plastic additive components of PM_2.5_ is closely associated with the prolongation of heart rate–corrected QT interval. Our findings highlight the importance of airborne industrial chemicals to cardiovascular disease risks.

## Introduction

Human exposure to ambient fine particles, such as PM_2.5_ (particles < 2.5 µm in aerodynamic equivalent diameter), has been reported with close associations with cardiovascular morbidity and mortality (1–3). The Harvard Six Cities Study and related studies reported that each increase in PM_2.5_ by 10 µg m^−3^ was associated with an adjusted increased risk of all-cause mortality and cardiovascular mortality of 14 and 26%, respectively (4–6). In particular, the associations between mass concentrations of PM_2.5_ exposure and the prolongation of QT (the time between the initial deflection of the QRS complex to the end of the T wave) interval, an important marker of ventricular repolarization and a risk factor for cardiac arrhythmia and sudden health, have been well documented in populations of different ages (7, 8).

Other than the mass concentration, myriad chemical components of PM_2.5_ may represent additional health risks, but the potential effects have been poorly explored in general (9, 10). Indeed, global studies have reported a large variety of plastic additive (PA) chemicals in the atmospheric environment, even in the polar regions (11–13). PAs represent a vast variety of industrial chemicals, functioning as plasticizers, antioxidants, stabilizers, flame retardants, lubricants, or pigments in various types of commercial goods (14, 15). As the additives are not chemically bound with their host products, a portion may migrate from in-use or discarded goods into the environment (14, 16). Some of the volatile or semivolatile PAs may be associated with ambient fine particles, resulting in a high complexity of chemical components of PM_2.5_ and long-range transport to global environments (11, 17). Many of the PAs have been documented with various toxicological effects. For example, exposure to selected phthalate esters (PAEs), a group of ubiquitous plasticizers, could be associated with increased blood pressure in humans or induce cardiac disorders in mice (18–20). Organophosphate esters (OPEs), a group of plasticizers and flame retardants, could increase the risk of hypertension and atherosclerosis (21, 22). However, there is a lack of clear documentation on whether airborne particle–containing PAs, either as complex mixtures or as individual components, could increase the risks of cardiovascular diseases.

**Fig. 1.**
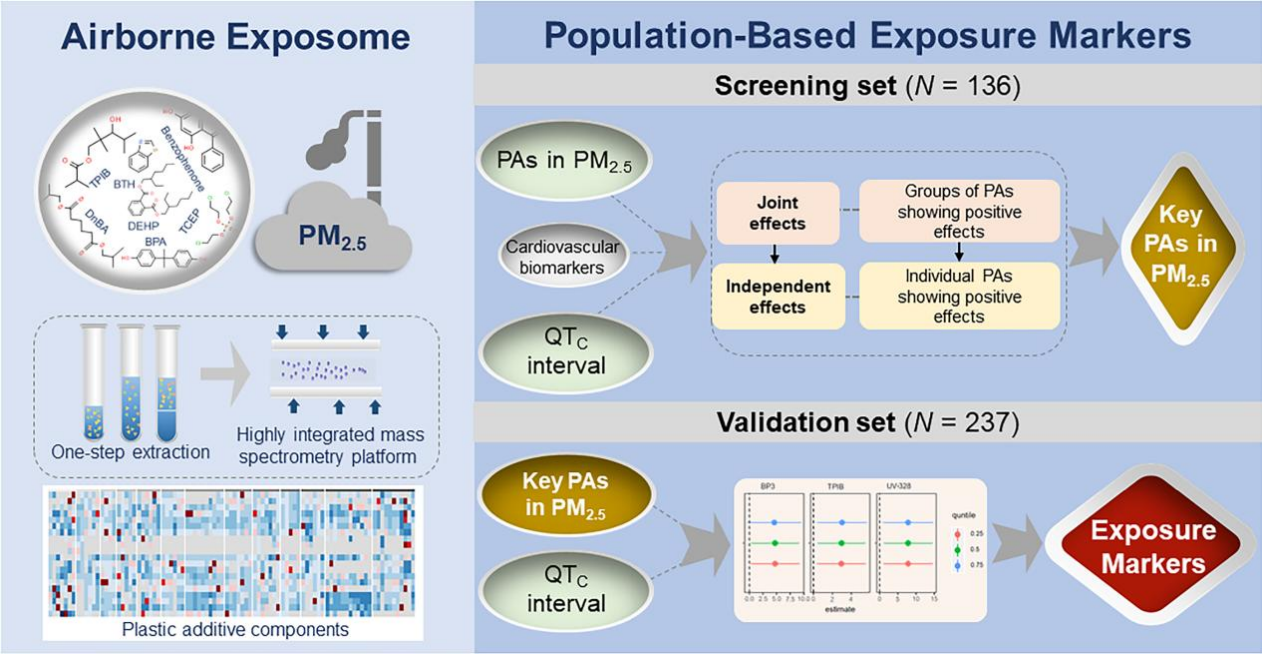
Flowchart of the study design.

**Fig. 2.**
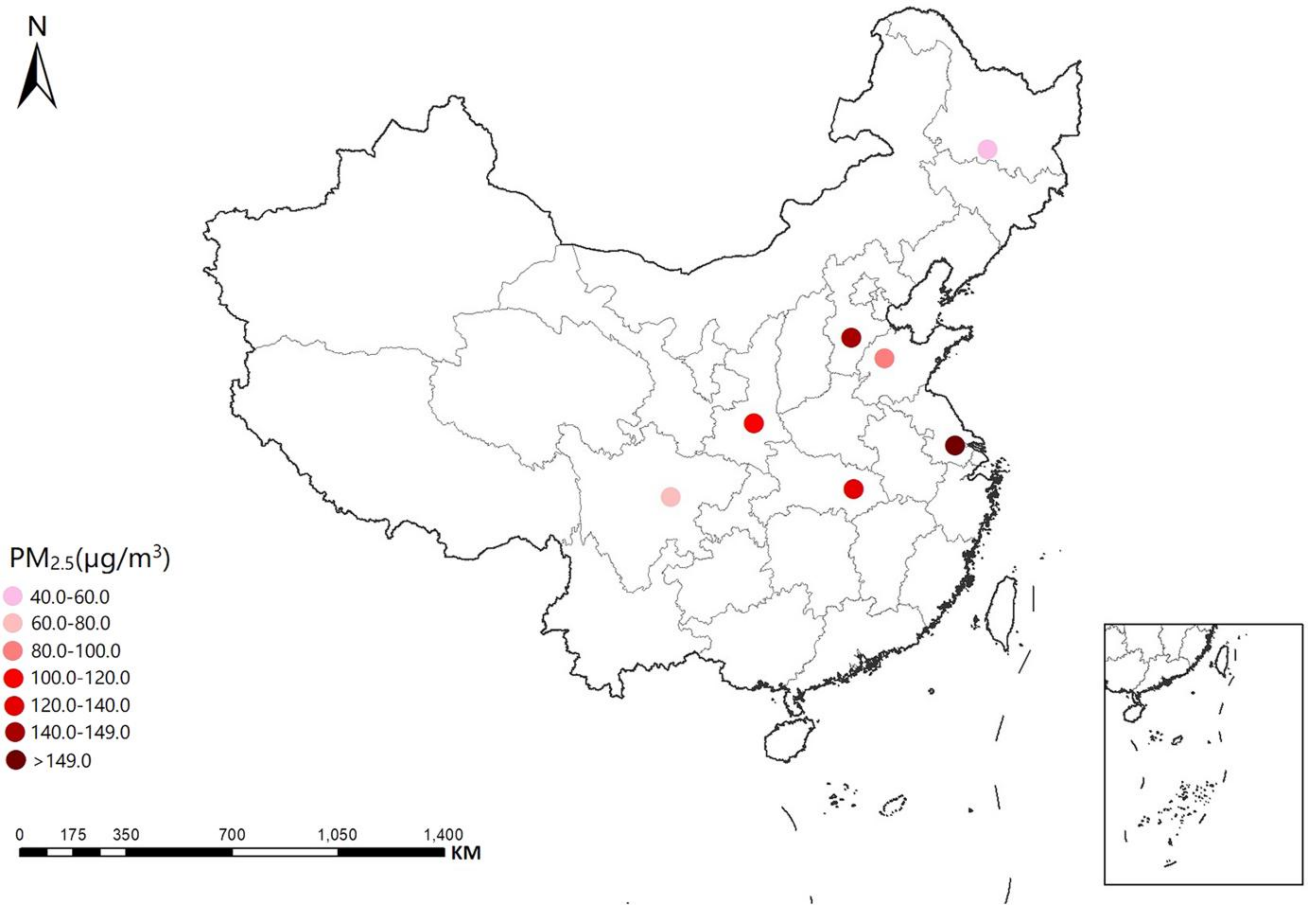
Study sites of the SCOPA-China cohort. The sites indicate seven communities located in Shijiazhuang (Hebei Province), Harbin (Heilongjiang Province), Wuhan (Hubei Province), Wuxi (Jiangsu Province), Jinan (Shandong Province), Xi’an (Shanxi Province), and Chengdu (Sichuan Province), respectively. The PM_2.5_ concentrations at each site are also shown.

Whether the complex PA components of PM_2.5_ acting alone or together represent additional cardiovascular risks constitutes an important public health question. To address this question, a high-throughput airborne exposome framework was developed in this study to quantitatively characterize PA components of PM_2.5_, and a population-based screening-to-validation strategy was employed to explore both the joint effects from the mixtures of PA components and the independent effects from individual PAs on heart rate–corrected QT (QT_C_) interval and selected cardiovascular biomarkers (Fig. 1). A total of 224 PAs were quantitatively measured in PM_2.5_ samples collected from 7 communities across China (Fig. 2), where a total of 373 participants aged between 40 and 88 were recruited through the Sub-Clinical Outcomes of Polluted Air in China (SCOPA-China) cohort (Tables S1 and S2). Key PAs would be identified as the exposure markers for the population health effect through the screening-to-validation strategy. Our work demonstrated for the first time the population-based link between exposure to PA components of PM_2.5_ and prolongation of QT_C_ interval. The findings highlighted the possible contributions of airborne industrial chemicals to cardiovascular disease burdens and underlined the critical need for further research on the underlying mechanisms, toxic modes of action, and human exposure risks.

## Results

### Airborne exposome characterization

**Fig. 3.**
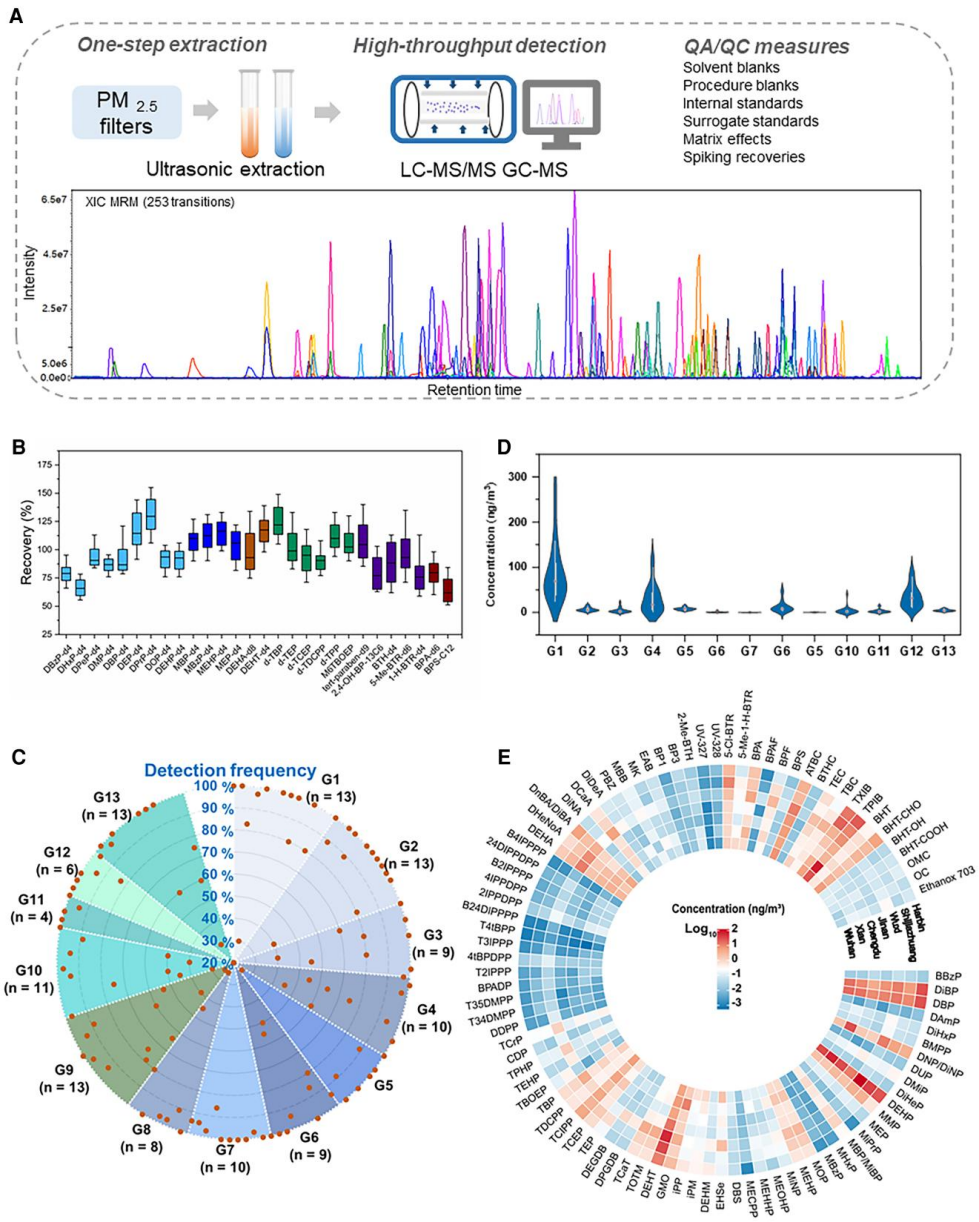
Concentrations of PAs by groups or individuals. A) A simplified flowchart showing the analytical procedures for the airborne exposome framework. B) Recovery data of isotopically labeled surrogate standards used for the analytical approach. C) A radar chart showing the detection frequency distributions of 127 PAs by groups (G1–G13) with a detection frequency >20% in PM_2.5_. D) A violin plot presenting the concentrations of PA components of PM_2.5_ by groups of chemicals. The data represent the combined concentrations of chemicals from each group. The box, center line, and vertical line at both ends represent the interquartile range, medium, and maximum and minimum of the data, respectively. The curved areas represent the distributions of concentration data. E) A circular heat map presenting the concentrations of individual PAs components of PM_2.5_. The data represent the median concentrations of individual chemicals at each study site (i.e. Wuhan, Xi’an, Chengdu, Jinan, Wuxi, Shijiazhuang, or Harbin; Fig. 2). Only chemicals with a detection frequency >80% are included. The full names of individual chemicals in the heat map are summarized in Table S2. G1: PAEs; G2: mono-PAEs; G3: FAE plasticizers; G4: other plasticizers; G5: alkyl-OPEs; G6: aryl-OPEs; G7: ITPs and TBPPs; G8: AEs; G9: BZPs and BZAs; G10: BTHs and BTRs; G11: BPs; G12: BEs and CEs; G13: SAOs.

Our target analytes included a total of 224 PAs that have been documented with broad industrial applications (Table S3). We developed a very efficient and high-throughput airborne exposome framework, including a one-step extraction strategy combined with a highly integrated mass spectrometry platform, to quantitatively determine the target PAs (Table S4). Data quality was ensured through a number of quality assurance and control practices (Fig. 3 and Table S5). Through the exposomic determination, 95 of the target PAs exhibited detection frequencies of >80% in our PM_2.5_ samples (Table S5), with the combined concentrations (referred to as ΣPAs) ranging from 66.3 to 555 ng m^−3^ across the 7 study sites. The concentrations varied greatly among individuals or different groups of chemicals (Fig. 3 and Table S6). The top five most abundant PAs, namely, di(2-ethylhexyl) phthalate (DEHP; median 28.8 ng m^−3^), di(2-ethylhexyl) terephthalate (DEHT; 16.5 ng m^−3^), dibutyl phthalate (15.3 ng m^−3^), diisobutyl phthalate (11.1 ng m^−3^), and 2,2,4-trimethyl-1,3-pentanediol-monoiso/butyrate (TPIB; 9.1 ng m^−3^), constituted 64% of the ΣPAs in the concentrations. By groups, PAEs (G1, median: 69.6 ng m^−3^) exhibited the highest levels measured in PM_2.5_, followed by butyrate and citrate esters (BEs and CEs; G12, 32.2 ng m^−3^), other plasticizers (G4, 17.0 ng m^−3^), alkyl-OPEs (G5, 8.05 ng m^−3^), adipate esters (AEs; G8, 7.81 ng m^−3^), phthalate mono-esters (mono-PAEs; G2, 5.45 ng m^−3^), synthetic antioxidants (SAOs; G13, 4.38 ng m^−3^), benzothiazoles and benzotriazoles (BTHs and BTRs; G10, 2.34 ng m^−3^), fatty acid ester (FAE) plasticizers (G3, 2.29 ng m^−3^), bisphenols (BPs; G11, 1.52 ng m^−3^), aryl-OPEs (G6, 0.54 ng m^−3^), benzophenones and benzoates (BZPs and BZAs; G9, 0.34 ng m^−3^), and isopropylated and *tert*-butylated triarylphosphate esters (ITPs and TBPPs; G7, 0.16 ng m^−3^). Our data demonstrate a high complexity of PAs with ubiquitous distributions in airborne particles.

### Joint effects of PA mixtures on QT_C_ interval

**Fig. 4.**
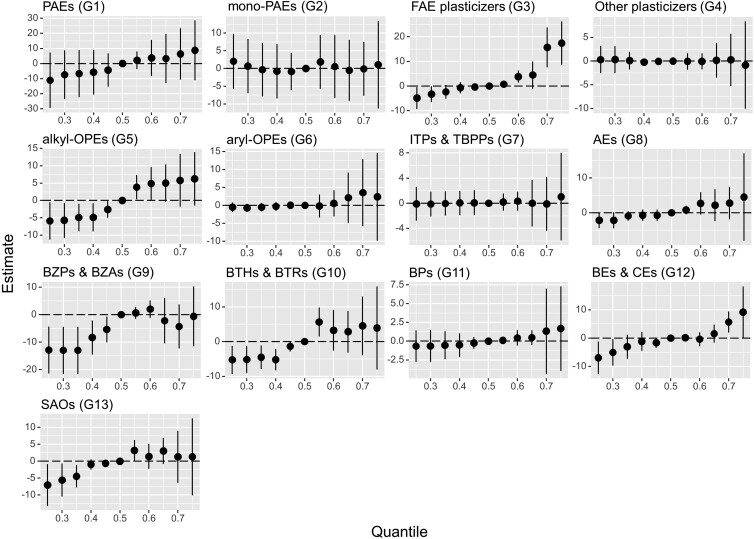
Joint effects of each group of PA components in PM_2.5_ on QT_C_ interval. The results were retrieved from the determination by the BKMR model with the data from the screening set. The full group names of the PAs are summarized in Fig. 3. The *x*-axis represents the percentile of the exposure levels of each group of chemicals (ranging from the 25th to the 75th percentile), and the *y*-axis represents the estimated change in the QT_C_ interval associated with a change in the exposure from a certain percentile when compared with when the exposure was at the median value (50th percentile).

The joint effects of exposure to each group of PAs on QT_C_ interval (estimates and 95% credible intervals, CIs) are summarized in Fig. 4. The graph represents the estimated change in the QT_C_ interval associated with a simultaneous change in the exposure levels of each component of the group from a particular threshold (25th to 75th percentile) when compared with when the components were each at the median value (50th percentile). Among the 13 groups of PAs, PAEs (G1), FAE plasticizers (G3), alkyl-OPEs (G5), aryl-OPEs (G6), AEs (G8), BZPs and BZAs (G9), BTHs and BTRs (G10), BPs (G11), BEs and CEs (G12), and SAOs (G13), all exhibited positive associations with the QT_C_ interval in the screening set. For example, the QT_C_ interval increased significantly when the exposure of the G3 group was at the 60th, 70th, or 75th percentile when compared with when the exposure was at the 50th percentile. Similarly, there was a significant increase in the QT_C_ interval when the exposure of the G5 group was at the 55th or 60th percentile when compared with when the exposure was at the 50th percentile, while the changes in the G10 and G13 exposure levels from the 50th percentile to the 55th percentile were both significantly associated with the increase in the QT_C_ interval.

### Independent effects and key PAs

**Fig. 5.**
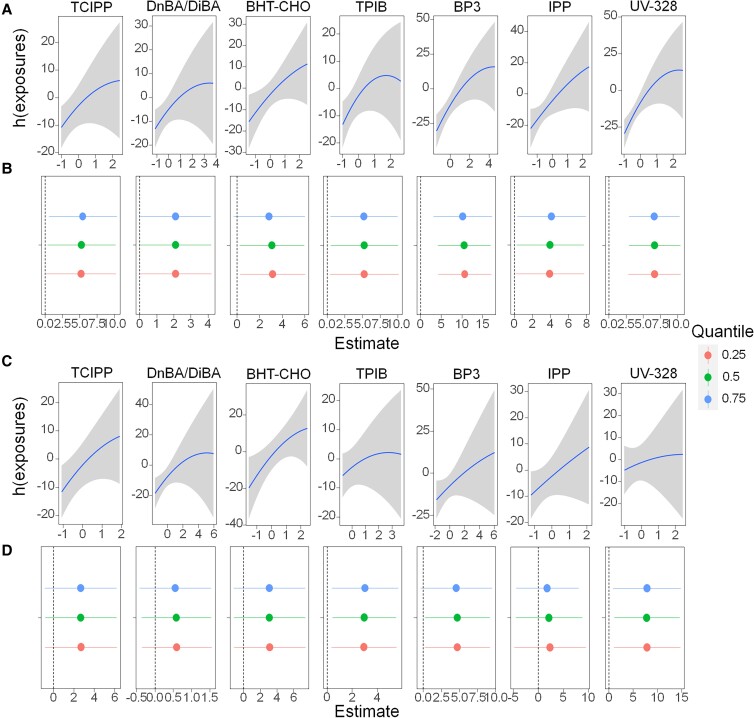
Independent effects of key PA components on QT_C_ interval. A and C) Univariate exposure–response functions and 95% CIs for individual key PAs, determined by the BKMR model in the screening set and validation set, respectively. The *x*-axis represents the exposure level of a single chemical after intragroup centralization, and the y-axis represents the estimated change in the QT_C_ interval associated with a change in a single chemical, when all of the other chemicals of the same group are fixed at the 50th percentile. B and D) Estimated change in the QT_C_ interval associated with a change in a single chemical from its 25th percentile to the 75th percentile, determined by the BKMR model in the screening set and validation set, respectively, where all of the other chemicals of the same group are fixed at a particular threshold (25th, 50th, or 75th percentile).

In addition to the joint effects explored above, the independent effects of individual PAs on QT_C_ interval were further determined. In the screening set, the univariate exposure–response function (and 95% CIs) indicated that the increased exposure to 8 of the 95 PAs in PM_2.5_ was significantly associated with the prolongation of the QT_C_ interval (Fig. 5A). These key PAs included tris(2-chloroisopropyl) phosphate (TCIPP), di-n-butyl adipate/diisobutyl adipate (DnBA/DiBA), 3,5-di-tert-butyl-4-hydroxybenzaldehyde (BHT-CHO), TPIB, 2-hydroxy-4-methoxybenzophenone (BP3), isopropyl palmitate (IPP), and 2-(2H-benzotriazol-2-yl)-4,6-di-tert-pentylphenol (UV-328). They belong to the PA groups of G3, G5, G8, G9, G10, G12, and G13, respectively.

To further explore the contribution of key individual PAs to the joint effects, Fig. 5B summarizes the estimated change in the QT_C_ interval associated with a change in a key PA from its 25th percentile to the 75th percentile, where all of the other chemicals of the same group were fixed at a particular threshold (25th, 50th, or 75th percentile). For instance, the changes in the QT_C_ interval were estimated to be 5.26 ms (95% CI: 0.36–10.16), 2.11 ms (95% CI: 0.03–4.18), 3.08 ms (95% CI: 0.25–5.92), 5.26 ms (95% CI: 0.53–9.98), 10.55 ms (95% CI: 4.20–16.90), 3.97 ms (95% CI: 0.26–7.70), and 6.64 ms (95% CI: 2.91–10.37), associated with a change in the exposure to TCIPP, DnBA/DiBA, BHT-CHO, TPIB, BP3, IPP, and UV-328 from their 25th percentile to the 75th percentile, respectively, when all of the other chemicals within each corresponding group were fixed at the 50th percentile. The effects of the key PAs on the QT_C_ interval did not change much when the remaining chemicals had concentrations fixed at the 75th, 50th, or 25th percentile. In short, the concentrations of the remaining chemicals in each group of PAs produced little influence on the independent effects by the key PAs.

Positive associations (although not statistically significant) between the above key PAs and most of the selected cardiovascular markers were also observed (Fig. S1). Eight cardiovascular markers, namely, α2-macroglobulin (A2M), adipsin, C-reactive protein (CRP), fetuin A, haptoglobin, platelet factor-4 (PF4), serum amyloid P (SAP), and von Willebrand factor (vWF), were positively associated with DnBA/DiBA, when the exposure from other chemicals within the same group was fixed at the 25th, 50th, or 75th percentile. Similarly, eight cardiovascular markers (i.e. A2M, adipsin, fetuin A, haptoglobin, L-selectin, PF4, SAP, and vWF) were positively associated with BHT-CHO, eight markers (i.e. A2M, adipsin, CRP, fetuin A, haptoglobin, PF4, SAP, and vWF) with TPIB and seven markers (i.e. A2M, adipsin, fetuin A, L-selectin, PF4, SAP, and vWF) with BP3. In addition, TCIPP was positively associated with haptoglobin, SAP, and vWF, while IPP was associated with A2M and fibrinogen. UV-328 was the only key PA not associated with any of the cardiovascular markers.

### Validation and identification of exposure markers

Both the joint effects of different groups of PAs and the independent effects of the key individual PAs on the QT_C_ interval were verified through the validation set. Among the 10 groups of PAs showing positive associations with the QT_C_ interval in the screening set, the positive trends remained in the validation set except for G10 (Fig. S2). Among the eight key PAs identified with significant associations with the prolongation of the QT_C_ interval during the screening phase, the significant associations remained for TCIPP, DnBA/DiBA, and BHT-CHO in the validation set. The changes in the QT_C_ interval were estimated to be 7.83 ms (95% CI: 1.09–14.59), 4.70 ms (95% CI: 0.22–9.19), and 2.99 ms (95% CI: 0.46–5.52), in association with a change in TCIPP, DnBA/DiBA, and BHT-CHO from their 25th percentile to the 75th percentile, respectively, when all of the other chemicals within each group were fixed at the 50th percentile (Fig. 5D). The other four chemicals (i.e. TPIB, BP3, IPP, and UV-328) also exhibited positive trends with the QT_C_ interval in the validation set, although the associations were no longer statistically significant. Therefore, both the joint effects by the mixtures of PAs and the independent effects by key individual PAs exhibited overall consistent patterns between the screening and the validation sets, indicating that our findings on the associations between exposure to the PA components of PM_2.5_ and prolongation of the QT_C_ interval are reproducible.

## Discussion

**Fig. 6.**
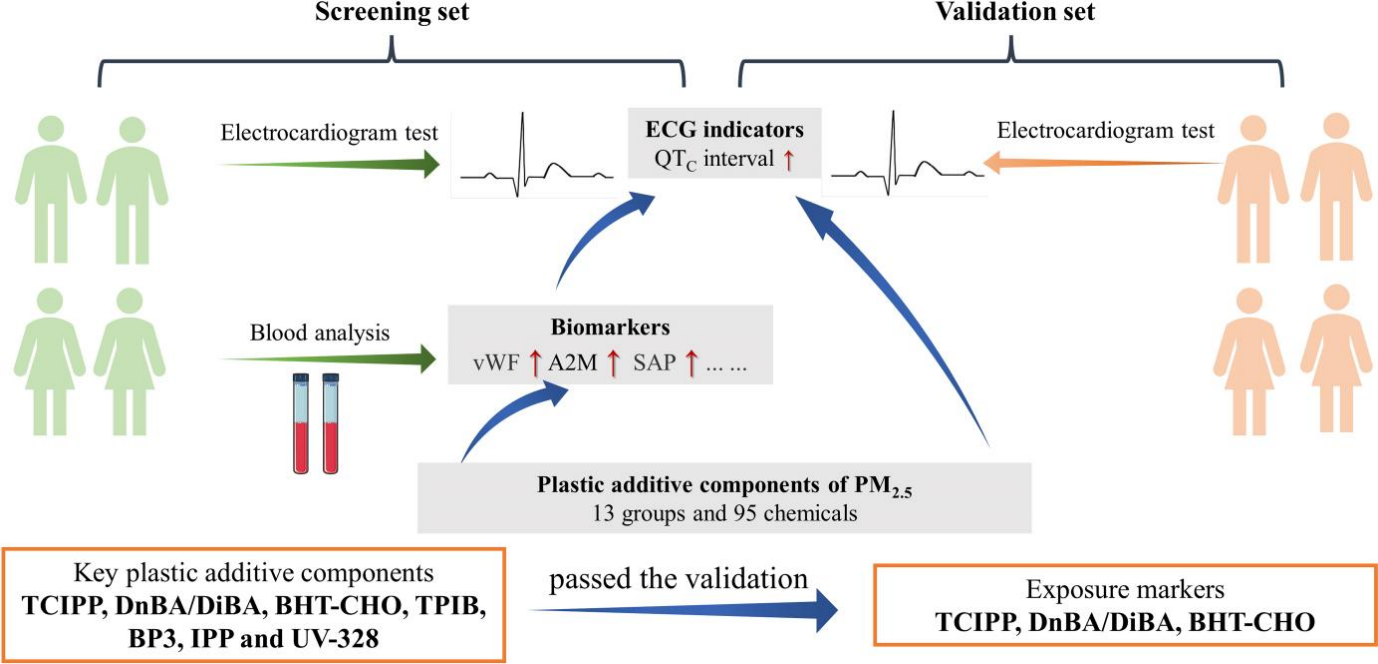
Summary of the main findings.

Our study demonstrated the impact of PA components of PM_2.5_ on the prolongation of the QT_C_ interval (Fig. 6). The airborne exposome analysis revealed ubiquitous occurrences of complex PAs in ambient environments, likely resulting from large-scale and long-term industrial applications. Joint effects from multiple mixtures of additive chemicals and independent effects from selected PAs on QT_C_ interval were identified and validated. Among the eight key PAs (i.e. TCIPP, DnBA/DiBA, BHT-CHO, TPIB, BP3, IPP, and UV-328) associated with QT_C_ interval prolongation and the possible adverse changes of cardiovascular biomarkers, TCIPP, DnBA/DiBA, and BHT-CHO were finally screened out as potential exposure markers for the population health effect (Fig. 6). In contrast, in our study, the PM_2.5_ concentrations did not exhibit a significant association with the QT_C_ interval in both the screening and the validation sets (Fig. S3).

The contributions of PA components of PM_2.5_ to human health risks in general and cardiovascular diseases in particular have not been sufficiently explored. The growing body of evidence from animal or epidemiological investigations has suggested that selected PAs could potentially increase the risk of cardiovascular diseases (23, 24). For example, exposure to selected PAEs and BP A was associated with major cardiovascular risk factors (e.g. hypertension, hyperlipemia, and myocardial infarction), as well as the risk of atherosclerosis and overt cardiovascular disease (24–26). Exposure to selected OPEs in vivo could induce cardiotoxicity and inhibit the cholesterol efflux in macrophages, resulting in the formation of foam cells and eventually leading to atherosclerosis (21). Epidemiological studies also reported the link between OPE exposure and hypertension and cardiotoxicity (21, 22). Indeed, our results indicated that 9 of the 13 groups of PAs exhibited positive trends with the QT_C_ interval in both the screening and the validation sets. As these chemicals are almost concurrently present in the ambient environment, their joint effects on cardiovascular health merit special concerns.

Our work strengthened the argument that exposure to airborne industrial chemicals could increase cardiovascular risks, as the prolongation of the QT_C_ interval is linked to a risk of developing potentially life-threatening ventricular tachyarrhythmias (27–29). In particular, TCIPP, BHT-CHO, and DnBA/DiBA, ranked as the 7th, 9th, and 10th most abundant PAs detected in PM_2.5_, respectively, can be used as exposure markers for the association with QT_C_ interval prolongation. These key PAs are representative of several important groups of PAs with broad industrial applications. For example, TCIPP represents one of the extensively used OPEs worldwide, mainly functioning as a flame retardant, adhesive agent, and plasticizer (30–32). DnBA and DiBA belong to the group of AEs and are widely used as plasticizers in personal care products (33, 34). BHT-CHO is one of the major transformation products of BHT, which as a SAO, finds broad applications in personal care products, cleaning products, and foodstuffs (35, 36). Large-scale applications of the key PAs have subsequently resulted in ubiquitous human exposure. Indeed, TCIPP, BHT-CHO, and BP3 have been detected in human blood or urine worldwide (35, 37–39). Human data remain limited for DnBA/DiBA, TPIB, IPP, and UV-328, most likely due to the lack of suitable biomonitoring markers, but these chemicals have already been demonstrated with widespread distributions in indoor and outdoor environments (11, 40, 41). Exposure to these key PAs could result in a prolongation of the QT_C_ interval generally within 10 ms in association with a change in the exposure from their 25th percentile to the 75th percentile. Such changes are not likely to induce polymorphic ventricular tachycardia in the clinical situation as long as the overall QT_C_ interval remains small (42). However, the coexistence of airborne exposure and other stressors (e.g. electrolyte disturbance or medication) could accentuate the lengthening of the QT_C_ interval. In short, these key PAs may allow us to efficiently assess the impacts of airborne exposure to PAs on QT_C_ interval and related cardiovascular diseases such as ventricular arrhythmia.

To date, there is a lack of information on the cause–effect relationships between exposure to the PA components of PM_2.5_ and the QT_C_ interval. We assumed that the exposure could disrupt selected cardiovascular biomarkers and prolongate the QT_C_ interval via a few possible pathways, including, but not limited to, disruption of cardiac potassium or sodium ion channels, induction of oxidative stress, and activation of calcium/calmodulin-dependent protein kinase II (CaMKII). Cardiac potassium ion channels are critical to maintaining the resting membrane potential and repolarization of the action potential in excitable cardiac myocytes (43). Some chemicals such as arsenic, PAHs, quinidine, and antibiotics have been reported to induce potassium current changes through blockade of the potassium channel, alterations of ion selectivity, or disruption of potassium channel gene expressions (e.g. *hERG*, human ether-a-go-go-related gene, or *KCNJ2*, potassium voltage–gated channel subfamily J member 2) (44, 45). Interferences of selected biomarkers such as CRP have also been reported to affect the QT_C_ interval through the mediation of the K^+^ channel interaction protein 2 (46). Direct effects on the electrophysiological properties of the heart, including the inhibition of sodium or potassium ion channels, have also been reported for selected PAs such as BP A, DEHP, and its metabolite mono-2-ethylhexyl phthalate, which subsequently causes a modulation of cardiac repolarization (47, 48). Oxidative stress is essentially involved in eliciting specific cardiac endpoints and modulating the risk of cardiovascular disease and metabolic disruptions following exposure to air pollution (49, 50). Previous studies have suggested that acute exposure to air pollution could promote cardiac arrhythmia, including a prolongation of the QT_C_ interval, through the mediation of oxidative stress (51, 52). Disruptions of several cardiovascular biomarkers such as adipsin, CRP, haptoglobin and fibrinogen have also been reported to be closely associated with oxidative stresses (53–55). Available studies have reported that exposure to TCIPP, BHT-CHO, BP3, UV-328, or di(2-ethylhexyl) adipate (structurally similar to DnBA) could increase reactive oxygen species generation and induce excessive oxidative stress (56–58), which would further impair endothelial-dependent vascular homeostasis (59). In addition, CaMKII activation has been reported to induce arrhythmias in structural diseases by modulation of several ion channels and transporters (60, 61). Exposure to high-level air pollution could induce arrhythmia in healthy mice via CaMKII activation as one of the possible mechanisms (59). Overall, future efforts are greatly needed to identify the underlying mechanisms through which airborne PAs would induce QT_C_ interval prolongation independently or as mixtures.

Our study presented several advantages. First, the study screened for a large complexity of PA components of PM_2.5_ and explored the associations with prolongation of the QT_C_ interval via a screening-to-validation strategy, ensuring that the findings and conclusions are solid. Second, our study employed a statistical method to assess both the joint effects of PA mixtures and the independent effects of individual chemical components of PM_2.5_. The study also considered potential nonlinear dose responses and interactions, although no significant outcomes were identified. Third, the study included multiple important cardiovascular biomarkers in the association analysis, which helps explore the potential mechanisms driving the influence of PA components of PM_2.5_ on QT_C_ interval and lays solid ground for further verification through experimental studies.

However, some limitations also existed in our study. First, the study conducted mediation analysis in the setting of a cross-sectional study design, which would be insufficient to capture the causal relationship between PAs in PM_2.5_ and QT_C_ interval. This limitation was partially counteracted by our validation strategy, which increased the weight of evidence. Second, there were significant differences in smoking and drinking status between the screening and the validation datasets, which might confound the analysis. However, the alcohol consumption and smoking habits as well as other basic information of participants were controlled during statistical analysis to exclude possible confounding effects. Third, a larger number of research participants than our current sample size would help reach more meaningful findings among the cardiovascular biomarkers measured, which will subsequently facilitate the exploration of potential mechanisms.

## Materials and methods

### Study design

As summarized in the flowchart of the study design (Fig. 1), a total of 373 participants were recruited from 7 communities through the SCOPA-China cohort (Table S1). A questionnaire survey, a physical examination, and biological sample collection were conducted for each participant. We measured 10 cardiovascular biomarkers in 136 participants, who constituted the screening set. The remaining 237 participants without cardiovascular biomarkers measured were included in the validation set in order to validate the associations between airborne exposure and QT_C_ interval prolongation and identify exposure markers for the population health effect (62). The average ages (±SD) of the screening and validation participants were 64.7 ± 12.9 and 63.4 ± 12.9 years old, respectively (Table S2). The screening and validation sets showed an average (±SD) body mass index (BMI) of 25.3 ± 4.0 and 25.6 ± 4.0 kg m^−2^ and an annual household income of (6.2 ± 5.7) × 10^4^ and (6.1 ± 6.9) × 10^4^ Chinese Yuan (CNY), respectively. The screening and validation sets exhibited no statistical differences in the average age, BMI, or annual income, as well as in the QT_C_ interval (i.e. 420.6 ± 30.2 vs. 423.1 ± 42.0 ms). The study was approved by the Ethics Committee of the National Institute of Environmental Health and Chinese Center for Disease Control and Prevention (201511, 201816). Informed consent was obtained from all participants.

### Electrocardiogram examination and cardiovascular biomarkers

An electrocardiogram (ECG) examination was conducted for all participants by trained medical staff. Briefly, the ECG was recorded for 5–10 min with a two-channel (12-lead) ECG monitor using a sampling rate of 256 Hz per channel. The QT interval was measured from each QRS onset to the end of the T wave for normal or supraventricular beats and corrected using Bazett's formula (62), and the data were automatically provided by the ECG moniter. The mean of the QT_C_ readings for the length of the recording was assigned to the corresponding participant's visit and expressed in milliseconds. If abnormal measurements (e.g. artifacts, U waves, bifid T waves, flat T waves, and arrhythmias) were encountered, the staff was instructed to perform repeated measurements after the participants took a brief rest. If abnormal measurements remained after repeated measurements, the data of the participants would be excluded from subsequent analysis.

Peripheral serum was collected from each participant. The participants were asked not to eat for 8 h prior to blood sampling. Serum samples were stored at −80°C prior to chemical analysis. For the participants in the screening set, 10 cardiovascular biomarkers were measured in serum by using the MILLIPLEX MAP Human Cardiovascular Disease (Acute Phase) Magnetic Bead Panel 3-Cardiovascular Disease Multiplex Assay kit (Merck KGaA, Darmstadt, Germany). The biomarkers included A2M, adipsin, CRP, fetuin A, fibrinogen, L-selectin, SAP, haptoglobin, PF4, and vWF (Table S7).

### PM_2.5_ sampling

PM_2.5_ sampling was conducted at each community with a moderate-volume sampler (TH-150C Intelligent PM_2.5_ sampler, Wuhan Tianhong Instrument Company, China) under the following conditions: (i) the sampling was conducted on the day of physical examination for each participant; (ii) the sampler was placed about 1.2 m above the rooftop to minimize the impact of dust suspension from the roof; (iii) there should be no major industrial sources within 5 km of the sampler; and (iv) each sampler was set up within 1–25 km radius among the recruited participants in each community. Before sampling, we set the zero point of the sampler and collected PM_2.5_ on a 90-mm diameter quartz filter (PALL, USA) at a flow rate of 100 L min^−1^. Prior to and after the sampling, the filters were balanced for at least 24 h in an environmental chamber and then weighted (XS105DU, Mettler Toledo Company, Switzerland). The collection of each PM_2.5_ sample took place for approximately 24 h and the sampling usually lasted for a total of 3–5 consecutive days at each location. For each participant, the concentrations of PA components in the 24-h sampling of PM_2.5_ collected during the day of physical examination were designated as his or her exposure levels.

### Exposomic characterization of PA components of PM_2.5_

A one-step extraction strategy was employed to extract target analytes from air filter. Each filter was cut into pieces and then placed into a 15-mL glass tube. After spiking with surrogate standards, the samples were extracted with 3 mL of acetonitrile in an ultrasonication bath for 15 min, followed with a mixture of hexane and dichloromethane (3 mL; v/v = 1:1) for 15 min. The supernatants were combined after centrifugation. The extraction was repeated twice and the combined extract was concentrated and then filtered (0.22 μm, VWR International). The final extract was concentrated, reconstituted with methanol, and then spiked with internal standards.

A highly integrated mass spectrometry platform was developed to quantitatively determine a total of 222 PAs based on an ultraperformance liquid chromatography coupled to a 5500 triple quadrupole mass spectrometry (AB Sciex, Toronto, Canada). DEHP and DEHT were determined on a gas chromatography coupled with 5977A single quadrupole mass (Agilent Technologies, Santa Clara, CA, USA). A total of 32 isotopically labeled chemicals were used as surrogate or internal standards. The detailed information on target analytes and instrumental analyses is summarized in Tables S3 and S4. Three chemicals coeluted with their corresponding isomers, and their concentrations were reported as a combination of coeluted ones (e.g. DnBA/DiBA represents the total concentration of DnBA and its isomer DiBA).

The limit of quantification (LOQ) of an analyte was defined as its response 10 times the standard deviation of the noise. For the chemicals detectable in blanks, their LOQs were determined as the average contamination in blanks plus 10 times the standard deviation of contamination (11). Several quality assurance and control procedures were conducted (see details in Table S5). Any analyte with >20% of concentration measurements below the LOQ was excluded from further exposure matching. PAs included in the statistical analysis were divided into 13 groups based on their molecular structures or application purposes (details given in Table S6).

### Covariate collection

The face-to-face questionnaire interview with the participants was conducted by our trained investigators. If there was any omission or logical error, the investigator would immediately supplement the data and provide feedback. The contents of the questionnaire were controlled as covariates, mainly including the basic information: sex (male, female, categorical) and age (continuous); lifestyles: smoking (never smoking, past smoking, and current smoking, categorical) and alcohol consumption (current drinking: participants who drank more than once a month on average during the last 12 months, never drinking: other participants besides current drinking, categorical); and the socioeconomic factor: annual household income (continuous). During physical examinations, height and weight data were collected from each participant. The BMI (kg m^−2^) was calculated by dividing weight in kilograms by the square of height in meters, and controlled as a continuous variable. The daily average temperature and relative humidity data were collected from the nearest monitoring station of each community via the China Meteorological Administration data sharing service (http://data.cma.cn), and controlled as continuous variables.

### Statistical analysis

Continuous variables were presented as mean ± SD, while the categorical variables were presented as numbers (percentage). To determine whether there is a significant difference between the descriptive statistics of the screening and validation sets, we used χ^2^ tests for categorical variables and Wilcoxon tests for nonnormally distributed continuous variables. The identification of key exposure markers from the complexity of PA components of PM_2.5_ included the three following steps.

#### Step 1: determine joint and independent effects and identify key PAs

The mixed exposure model Bayesian kernel machine regression (BKMR) was used to establish the associations of 13 groups of PAs with the QT_C_ interval in the screening set. The BKMR model allows for identifying independent effects from individual exposure in addition to the joint effects from exposure to a mixture of chemicals by using a kernel function (63, 64). The exposure levels in each group were centralized, i.e. subtracting the mean of the concentration matrix and dividing it by the standard deviation of the concentration matrix for each group. We created the BKMR models based on the following equation for each of the 13 groups of PAs:


(1) $$Y_{i}=h(Z)+\beta z_{i}+e_{i}$$

where *Y_i_* is the QT_C_ interval; *h ()* is the exposure–response function, which incorporates both nonlinear relationships and interactions among exposures, with the concentrations of PAs treated as *Z* to fit the model; *z_i_* is a vector of covariates, including sex, age, BMI, income, smoking, drinking, temperature, and relative humidity; *e_i_* is a random error term. We used a Gaussian link function with BKMR in consideration of the current continuous outcome and implemented a hierarchical variable selection method with 50,000 iterations using a Markov chain Monte Carlo algorithm. Key PAs could be screened out when the overall effect of the group of chemicals containing the key PAs exhibited a positive association trend with QT_C_, while these individual chemicals were also significantly associated with the prolongation of the QT_C_ interval.

#### Step 2: establish the associations between key PAs and cardiovascular biomarkers

The BKMR model was used to further analyze the associations between the key individual PAs significantly associated with the QT_C_ interval (identified in step 1) and 10 cardiovascular biomarkers. The same covariates were adjusted, and the same number of iterations were applied to the BKMR model.

#### Step 3: validate the key PAs as exposure markers for the population health effect

To verify the results of the screening set, we used the validation set to establish the BKMR model (Eq. 1) between the QT_C_ interval and the identified key PAs. If the key PAs remained with significant associations with the prolongation of the QT_C_ interval in the validation set, the result of the screening phase was considered to pass the verification test.

## Supplementary Material

pgad397_Supplementary_Data

## Data Availability

All data are available in the main text or the Supplementary material.
