# Supplementary material for: Plastic additive components of PM2.5 increase corrected QT interval: Screening for exposure markers based on airborne exposome
Source: PNAS Nexus. 2023 Nov 23;2(12):pgad397. doi: 10.1093/pnasnexus/pgad397 (PMC10691654; doi:10.1093/pnasnexus/pgad397)
Supplement: pgad397_Supplementary_Data [file pgad397_supplementary_data.docx]

**
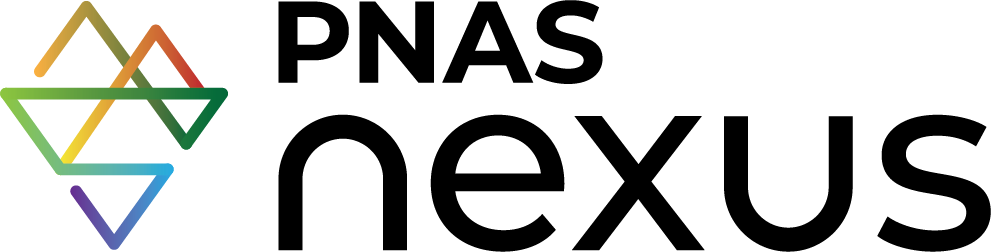
**

**Supplementary Information for**

**Plastic Additive Components of PM_2.5_ Increase Corrected QT Interval: Screening for Exposure Markers Based on Airborne Exposome**

Xiaotu Liu^1^, Yanwen Wang^2^, Jianlong Fang^2^, Renjie Chen^3^, Yue Sun^2^, Shuqin Tang^1^, Minghao Wang^2^, Haidong Kan^3^, Tiantian Li^2,^*, Da Chen^1,^*

^1^School of Environment and Guangdong Key Laboratory of Environmental Pollution and Health, Jinan University, Guangzhou 511443, China

^2^China CDC Key Laboratory of Environment and Population Health, National Institute of Environmental Health, Chinese Center for Disease Control and Prevention, Beijing, 100021, China

^3^School of Public Health, Key Lab of Public Health Safety of the Ministry of Education and NHC Key Lab of Health Technology Assessment, Shanghai Institute of Infectious Disease and Biosecurity, Fudan University, Shanghai 200032, China

*Da Chen, Tiantian Li

**Email:**  [dachen@jnu.edu.cn](mailto:dachen@jnu.edu.cn), [litiantian@nieh.chinacdc.cn](mailto:litiantian@nieh.chinacdc.cn)

**This PDF file includes:**

Figures S1 to S3

Tables S1 to S7


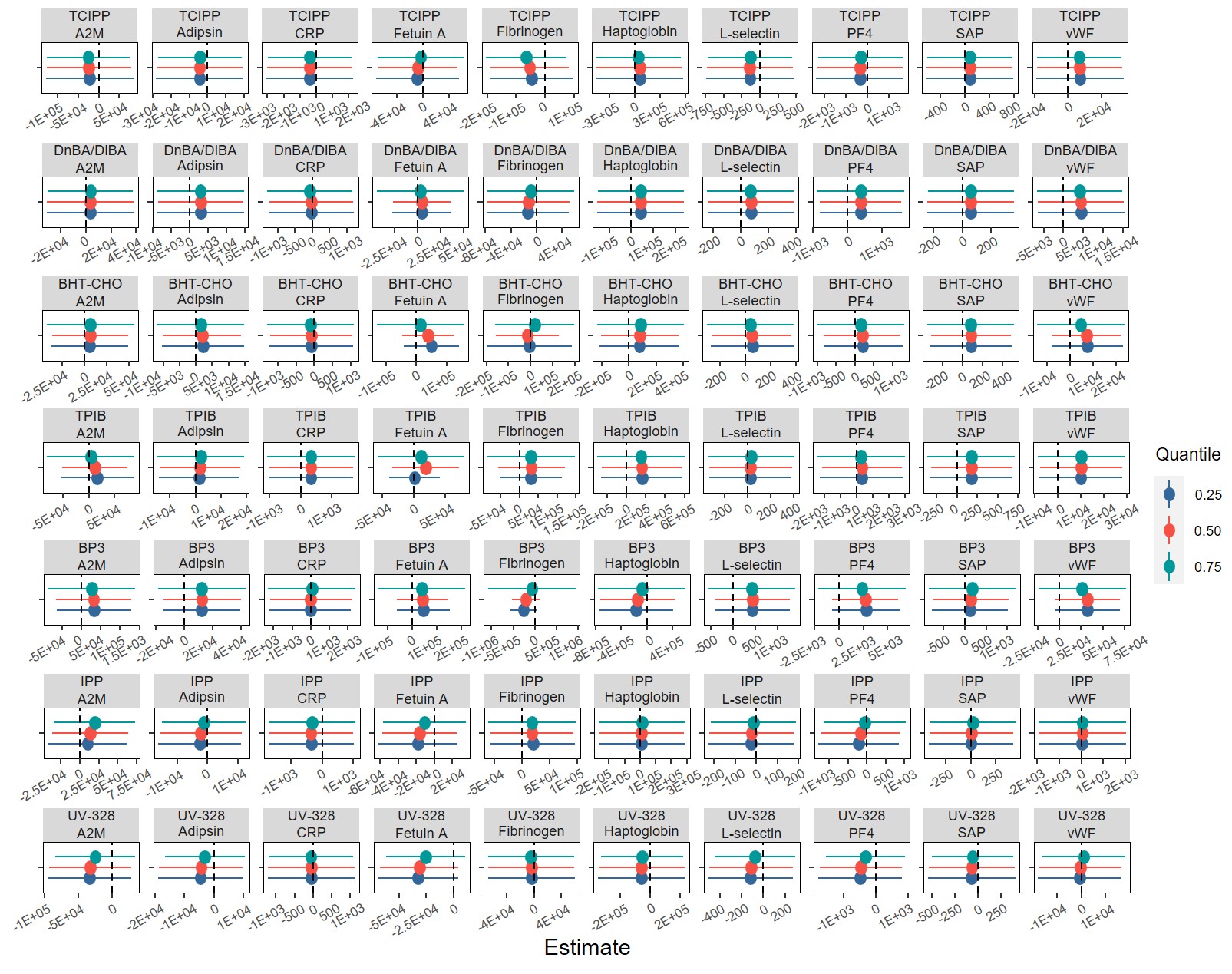


Figure S1. Independent effects (95% credible intervals, CIs) of selected plastic additive. The effects of individual plastic additive components of PM2.5 on cardiovascular biomarkers were determined by using the BKMR models. TCIPP: tris(2-chloroisopropyl) phosphate; DnBA/DiBA: di-n-butyl adipate/diisobutyl adipate; BHT-CHO: 3,5-di-tert-butyl-4-hydroxybenzaldehyde; TPIB: 2,2,4-trimethyl-1,3-pentanediol-monoisobutyrate; BP3: 2-hydroxy-4-methoxybenzophenone; IPP: isopropyl palmitate; UV-328: 2-(2H-benzotriazol-2-yl)-4,6-di-tert-pentylphenol. A2M: α2-macroglobulin; CRP: C-reactive protein; PF4: platlet factor 4; SAP: serum amyloid P; vWF, von Willebrand factor.


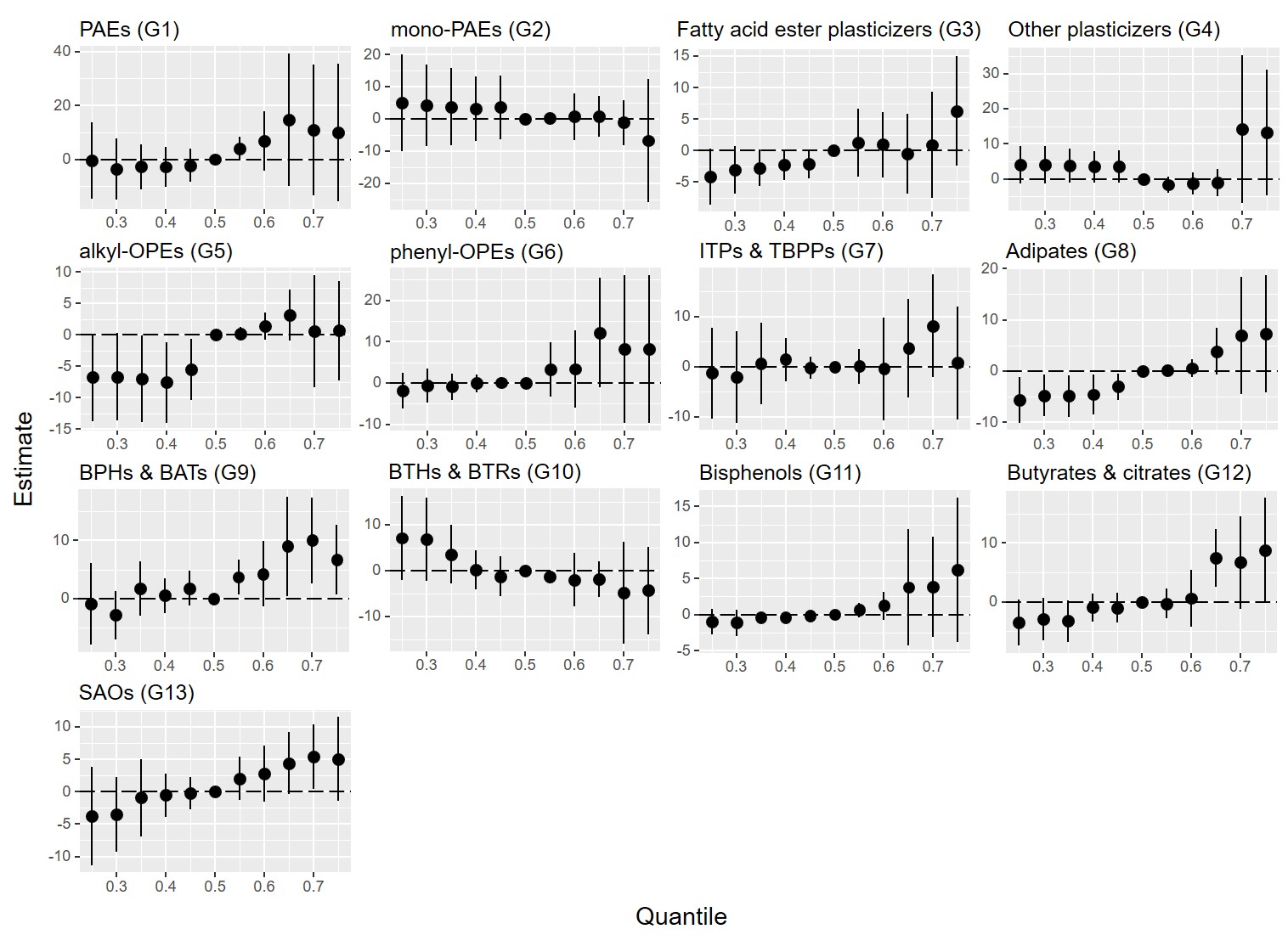


Figure S2. Joint effects of each group of plastic additive components in PM_2.5_ on QT_C_ interval. The results were retrieved from the determination by the *BKMR* model with the data from the validation set. The *x*-axis represents the percentile of the exposure levels of each group of chemicals (ranging from the 25^th^ to 75 percentile), and the *y*-axis represents the estimated change in QT_C_ interval associated with a change of the exposure from a certain percentile as compared to when the exposure was at the median value (50^th^ percentile). G1: phthalate esters (PAEs); G2: phthalate mono-esters (mono-PAEs); G3: fatty acid ester (FAE) plasticizers; G4: other plasticizers; G5: alkyl organophosphate esters (alkyl-OPEs); G6: aryl organophosphate esters (aryl-OPEs); G7: isopropylated and *tert*-butylated triarylphosphate esters (ITPs & TBPPs); G8: adipate esters (AEs); G9: benzophenones and benzoates (BZPs & BZAs); G10: benzothiazoles and benzotriazoles (BTHs & BTRs); G11: bisphenols (BPs); G12: butyrate and citrate esters (BEs & CEs); G13: synthetic antioxidants (SAOs).


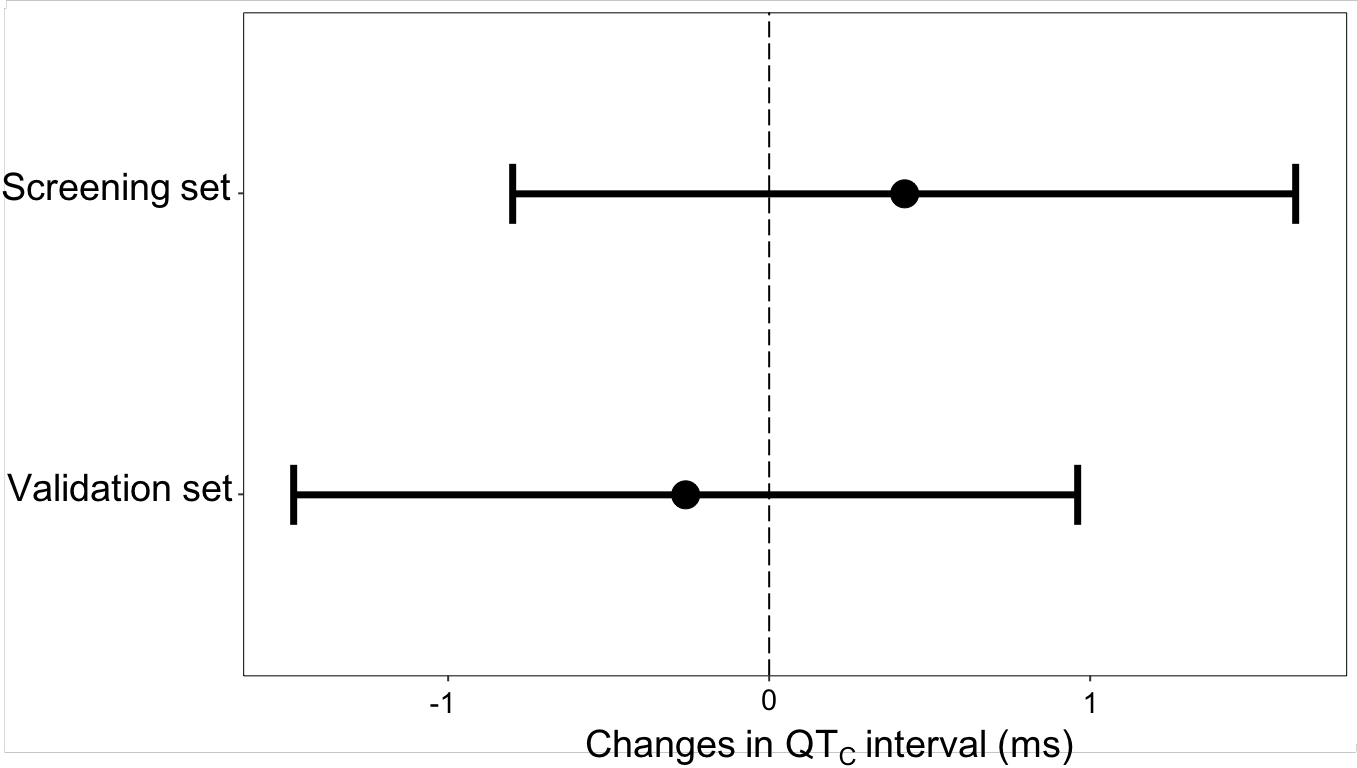


Figure S3. Changes in QTc interval (ms) for every 10 μg/m3 increase in PM2.5 concentrations.

**Table S1.** The number (and percentage) of human subjects recruited at each investigation site, including the screening and validation sets.

| **Province** | **City** | **Total**  **(N = 373)** | **Screening set**  **(N = 136)** | **Validation set**  **(N = 237)** |
| --- | --- | --- | --- | --- |
| Hebei | Shijiazhuang | 58 (15.6%) | 20 (14.7%) | 38 (16.0%) |
| Heilongjiang | Harbin | 46 (12.3%) | 24 (17.7%) | 22 (9.3%) |
| Hubei | Wuhan | 20 (5.4%) | / **^a^** | 20 (8.4%) |
| Jiangsu | Wuxi | 133 (35.7%) | 55 (40.4%) | 78 (32.9%) |
| Shandong | Jinan | 97 (26.0%) | 30 (22.1%) | 67 (28.3%) |
| Shaanxi | Xi'an | 12 (3.2%) | / | 12 (5.1%) |
| Sichuan | Chengdu | 7 (1.9%) | 7 (5.2%) | / |

**^a^** No subjects recruited.

Table S2. Summary of the participant characteristics in the screening (*N* = 136) and validation set (*N* = 237) ^a^.

|  | **Screening set**  **(*N*=136)** | **Validation set**  **(*N*=237)** | ***p*-value^b^** |
| --- | --- | --- | --- |
| Gender |  |  |  |
| Male | 59 (43.4%) | 121 (51.0%) | 0.19 |
| Female | 77 (56.6%) | 116 (49.0%) |  |
| Age (years) | 64.7±12.9 | 63.4 ± 12.9 | 0.33 |
| BMI**^c^** (kg/m^2^) | 25.3±4.0 | 25.6 ± 4.0 | 0.73 |
| Income (10^4^ CNY/year)**^d^** | 6.2±5.7 | 6.1 ± 6.9 | 0.86 |
| Drinking |  |  |  |
| Never drinking | 110 (80.9%) | 154 (65.0%) | <0.05 |
| Current drinking | 22 (16.2%) | 83 (35.0%) |  |
| Unclear | 4 (2.9%) | 0 (0.0%) |  |
| Smoking |  |  |  |
| Never smoking | 117 (86.0%) | 120 (50.6%) | <0.05 |
| Past smoking | 7 (5.2%) | 33 (13.9%) |  |
| Current smoking | 12 (8.8%) | 84 (35.4%) |  |
| QT_C_ (ms) | 420.6±30.2 | 423.1 ± 42.0 | 0.42 |
| Temperature (℃) | 13.5±1.9 | 13.1 ± 3.1 | 0.54 |
| Relative Humidity (%) | 71.7±11.0 | 73.0 ± 11.1 | 0.12 |

**^a^** Continuous variables are presented as mean ± SD. Category variables are presented as numbers (percentage).

**^b^** The Wilcoxon test and Chi-square test were employed for non-normally distributed continuous variables and categorical variables, respectively. The level of significance was set at *p* < 0.05.

**^c^** BMI, body mass index.

**^d^** CNY, Chinese Yuan, 1 CNY ≈ 0.16 US Dollars.

**Table S3.** Full names, commercial names or abbreviations, CAS numbers, suppliers, corresponding surrogate standards, and quantification methods of target plastic additives, as well as the information of the reagents used in the study.

| **Target plastic additives** | | | | | |
| --- | --- | --- | --- | --- | --- |
| **Commercial name or abbreviation** | **Full Name** | **CAS number** | **Supplier** | **Surrogate standards** | **Quantification methods^a^** |
| BBzP | benzyl butyl phthalate | 85-68-7 | AccuStandard | DBzP-d4 | PA-ESI |
| iBCHP | isobutylcyclohexyl phthalate | 5334-09-8 | AccuStandard | DHxP-d4 | PA-ESI |
| BMPP | bis(4-methyl-2-pentyl) phthalate | 146-50-9 | AccuStandard | DPeP-d4 | PA-ESI |
| DAlP | diallyl phthalate | 131-17-9 | AccuStandard | DBzP-d4 | PA-ESI |
| DAmP | diamyl phthalate | 131-18-0 | AccuStandard | DMP-d4 | PA-ESI |
| DiBP | diisobutyl phthalate | 84-69-5 | AccuStandard | DBP-d4 | PA-ESI |
| DBP | dibutyl phthalate | 84-74-2 | AccuStandard | DBP-d4 | PA-ESI |
| DBzP | dibenzyl phthalate | 523-31-9 | AccuStandard | DBzP-d4 | PA-ESI |
| DEP | diethyl phthalate | 84-66-2 | AccuStandard | DEP-d4 | PA-ESI |
| DEHP | di(2-ethylhexyl) phthalate | 117-81-7 | AccuStandard | DEHP-d4 | GC |
| DiHeP | diisoheptyl phthalate | 71888-89-6 | AccuStandard | DPeP-d4 | PA-ESI |
| DiHxP | diisohexyl phthalate | 68515-50-4 | AccuStandard | DHxP-d4 | PA-ESI |
| DHxP | dihexyl phthalate | 84-75-3 | AccuStandard | DHxP-d4 | PA-ESI |
| DMiP | dimethyl isophthalate | 1459-93-4 | AccuStandard | DMP-d4 | PA-ESI |
| DMP | dimethyl phthalate | 131-11-3 | AccuStandard | DMP-d4 | PA-ESI |
| DiNP | diisononyl phthalate | 68515-48-0 | AccuStandard | DOP-d4 | PA-ESI |
| DNP | dinonyl phthalate | 84-76-4 | AccuStandard | DOP-d4 | PA-ESI |
| DiPeP | diisopentyl phthalate | 605-50-5 | AccuStandard | DPeP-d4 | PA-ESI |
| DPHiP | diphenyl isophthalate | 744-45-6 | AccuStandard | DPeP-d4 | PA-ESI |
| DPHP | diphenyl phthalate | 84-62-8 | AccuStandard | DPeP-d4 | PA-ESI |
| DiPrP | diisopopyl phthalate | 605-45-8 | AccuStandard | DPrP-d4 | PA-ESI |
| DPrP | dipopyl phthalate | 131-16-8 | AccuStandard | DPrP-d4 | PA-ESI |
| DUP | diundecyl phthalate | 3648-20-2 | AccuStandard | DBzP-d4 | PA-ESI |
| MiBP | Monoisobutyl phthalate | 30833-53-5 | AccuStandard | MBP-d4 | PA-ESI |
| MBP | Monobutyl phthalate | 131-70-4 | AccuStandard | MBP-d4 | PA-ESI |
| MBzP | Monobenzyl phthalate | 2528-16-7 | AccuStandard | MBzP-d4 | PA-ESI |
| MCHP | Monocyclohexyl phthalate | 7517-36-4 | AccuStandard | MBP-d4 | PA-ESI |
| MEP | Monoethyl phthalate | 2306-33-4 | AccuStandard | MEP-d4 | PA-ESI |
| MEHP | Monoethylhexyl phthalate | 4376-20-9 | AccuStandard | MEHP-d4 | PA-ESI |
| MECPP | Mono (2-ethyl-5carboxypentyl) phthalate | 40809-41-4 | TRC **^b^** | MEHP-d4 | PA-ESI |
| MEHHP | Mono (2-ethyl-5-hydroxyhexyl) phthalate | 40321-99-1 | TRC | MEHP-d4 | PA-ESI |
| MEOHP | Mono (2-ethyl-5-oxohexyl) phthalate | 40321-98-0 | TRC | MEHP-d4 | PA-ESI |
| MHeP | Mono-2-heptyl phthalate | 129171-03-5 | AccuStandard | MBzP-d4 | PA-ESI |
| MHxP | monohexyl phthalate | 24539-57-9 | AccuStandard | MBP-d4 | PA-ESI |
| MMP | Monomethyl phthalate | 4376-18-5 | AccuStandard | MEP-d4 | PA-ESI |
| MiNP | Monoisononyl phthalate | 106610-61-1 | AccuStandard | MEHP-d4 | PA-ESI |
| MOP | Monooctyl phthalate | 5393-19-1 | AccuStandard | MBzP-d4 | PA-ESI |
| MPeP | Monopentyl phthalate | 24539-56-8 | AccuStandard | MBP-d4 | PA-ESI |
| MiPrP | Monoisopropyl phthalate | 35118-50-4 | AccuStandard | MEP-d4 | PA-ESI |
| BARO | butyl acetyl ricinoleate | 140-04-5 | AccuStandard | DEHA-d8 | PA-ESI |
| BRO | butyl ricinoleate | 151-13-3 | AccuStandard | DEHA-d8 | PA-ESI |
| BO | polycizer butyl oleate | 142-77-8 | AccuStandard | DEHA-d8 | PA-ESI |
| DBF | dibutyl fumarate | 105-75-9 | AccuStandard | DEHA-d8 | PA-ESI |
| DBS | dibutyl sebacate | 109-43-3 | AccuStandard | DEHA-d8 | PA-ESI |
| DEHM | di(2-ethylhexyl) maleate | 142-16-5 | AccuStandard | DEHA-d8 | PA-ESI |
| DES | diethyl succinate | 123-25-1 | AccuStandard | DEHA-d8 | PA-ESI |
| DMS | dimethyl sebacate | 106-79-6 | AccuStandard | DEHA-d8 | PA-ESI |
| EHSe | 2-ethylhexyl sebacate | 122-62-3 | AccuStandard | DEHA-d8 | PA-ESI |
| GMO | glycerol monooleate | 25496-72-4 | AccuStandard | DEHA-d8 | PA-ESI |
| MO | methyl oleate | 112-62-9 | AccuStandard | DEHA-d8 | PA-ESI |
| MOA | methyl O-acetylricinoleate | 140-03-4 | AccuStandard | DEHA-d8 | PA-ESI |
| IPM | isopropyl myristate | 110-27-0 | AccuStandard | DEHA-d8 | PA-ESI |
| IPP | isopropyl palmitate | 142-91-6 | AccuStandard | DEHA-d8 | PA-ESI |
| PO | propyl oleate | 111-59-1 | AccuStandard | DEHA-d8 | PA-ESI |
| THFO | tetrahydrofurfuryl oleate | 5420-17-7 | AccuStandard | DEHA-d8 | PA-ESI |
| DEHT | di(2-ethylhexyl) terephthalate | 6422-86-2 | AccuStandard | DEHT-d4 | GC |
| DEGDB | diethylene glycol, dibenzoate | 120-55-8 | AccuStandard | DEHA-d8 | PA-ESI |
| DPGDB | dipropylene glycol, dibenzoate | 27138-31-4 | AccuStandard | DEHA-d8 | PA-ESI |
| DINCH | di-isononyl cyclohexane-1,2-dicarboxylate | 166412-78-8 | TRC | DEHA-d8 | PA-APCI |
| DiDEAZ | diisodecyl azelate | 28472-97-1 | AccuStandard | DEHA-d8 | PA-APCI |
| DHAZ | di-n-hexyl azelate | 109-31-9 | AccuStandard | DEHA-d8 | PA-ESI |
| DMAZ | dimethyl azelate | 1732-10-1 | AccuStandard | DEHA-d8 | PA-ESI |
| DiOAZ | diisooctyl azelate | 26544-17-2 | AccuStandard | DEHA-d8 | PA-ESI |
| TCaT | tricapryl trimellitate | 27251-75-8 | AccuStandard | DEHA-d8 | PA-ESI |
| THTM | trihexyl trimellitate | 1528-49-0 | AccuStandard | DEHA-d8 | PA-ESI |
| TiNTM | triisononyl trimellitate | 53894-23-8 | AccuStandard | DEHA-d8 | PA-ESI |
| TOTM | trioctyl trimellitate | 3319-31-1 | AccuStandard | DEHA-d8 | PA-ESI |
| TiTDM | triisodecyl trimelliate | 36631-30-8 | AccuStandard | DEHA-d8 | PA-ESI |
| TBP | tributyl phosphate | 126-73-8 | AccuStandard | d-TBP | PA-ESI |
| TBOEP | tris(2-butoxyethyl) phosphate | 78-51-3 | AccuStandard | M6TBOEP | PA-ESI |
| TCEP | tris(2-chloroethyl) phosphate | 115-96-8 | AccuStandard | d-TCEP | PA-ESI |
| TCIPP | tris(2-chloroisopropyl) phosphate | 13674-84-5 | AccuStandard | d-TDCPP | PA-ESI |
| TDBPP | tris(2,3-dibromopropyl) phosphate | 126-72-7 | AccuStandard | d-TDCPP | PA-ESI |
| TDCPP | tris(1,3-dichloro-2-propyl) phosphate | 13674-87-8 | AccuStandard | d-TDCPP | PA-ESI |
| TEP | triethyl phosphate | 78-40-4 | AccuStandard | d-TEP | PA-ESI |
| TEHP | tris(2-ethylhexyl) phosphate | 78-42-2 | AccuStandard | d-TBP | PA-ESI |
| TPrP | tripropyl phosphate | 513-08-6 | AccuStandard | d-TCEP | PA-ESI |
| V6 | tetrakis(2-chloroethyl)dichloroisopentyl diphosphate | 38051-10-4 | AccuStandard | M6TBEP | PA-ESI |
| BPADP | bisphenol A bis(diphenyl phosphate) | 5945-33-5 | AccuStandard | d-TPP | PA-ESI |
| CDP | cresyl diphenyl phosphate | 26444-49-5 | AccuStandard | d-TPP | PA-ESI |
| DDPP | isodecyl diphenyl phosphate | 29761-21-5 | AccuStandard | d-TPP | PA-ESI |
| EHDPP | 2-ethylhexyl-diphenyl phosphate | 1241-94-7 | AccuStandard | d-TBP | PA-ESI |
| RDP | resorcinol bis(diphenyl phosphate) | 57583-54-7 | AccuStandard | d-TPP | PA-ESI |
| TCrP | tricresyl phosphate | 1330-78-5 | AccuStandard | d-TPP | PA-ESI |
| T34DMPP | tris(3,4-dimethyphenyl) phosphate | 3862-11-1 | Wellington | d-TPP | PA-ESI |
| T35DMPP | tris(3,5-dimethylphenyl) phosphate | 25653-16-1 | Wellington | d-TPP | PA-ESI |
| TPHP | triphenyl phosphate | 115-86-6 | AccuStandard | d-TCEP | PA-ESI |
| B2tBPPP | bis(2-tert-butylphenyl) phenyl phosphate | 65652-41-7 | Wellington | d-TPP | PA-ESI |
| B4tBPPP | bis(4-tert-butylphenyl) phenyl phosphate | 115-87-7 | Wellington | d-TPP | PA-ESI |
| 2tBPDPP | 2-tert-butylphenyl diphenyl phosphate | 83242-23-3 | Wellington | d-TPP | PA-ESI |
| 4tBPDPP | 4-tert-butylphenyl diphenyl phosphate | 981-40-8 | Wellington | d-TPP | PA-ESI |
| 24DIPPDPP | 2,4-diisopropylphenyl diphenyl phosphate | 96107-55-0 | Wellington | d-TPP | PA-ESI |
| B24DIPPPP | bis(2,4-diisopropylphenyl) phenyl phosphate | 2190501-29-0 | Wellington | d-TPP | PA-ESI |
| B2IPPPP | bis(2-isopropylphenyl) phenyl phosphate | 69500-29-4 | Wellington | d-TPP | PA-ESI |
| B4IPPPP | bis(4-isopropylphenyl) phenyl phosphate | 55864-07-8 | Wellington | d-TPP | PA-ESI |
| 2IPPDPP | 2-isopropylphenyl diphenyl phosphate | 64532-94-1 | Wellington | d-TPP | PA-ESI |
| 4IPPDPP | 4-isopropylphenyl diphenyl phosphate | 55864-04-5 | Wellington | d-TPP | PA-ESI |
| T4tBPP | tris (4-tert-butylphenyl) phosphate | 78-33-1 | Wellington | d-TPP | PA-ESI |
| T2IPPP | tris(2-isopropylphenyl) phosphate | 64532-95-2 | AccuStandard | d-TPP | PA-ESI |
| T3IPPP | tris (3-isopropylphenyl) phosphate | 72668-27-0 | Wellington | d-TPP | PA-ESI |
| T4IPPP | tris (4-isopropylphenyl) phosphate | 2502-15-0 | Wellington | d-TPP | PA-ESI |
| DiBA | diisobutyl adipate | 141-04-8 | TCI chemicals | DEHA-d8 | PA-ESI |
| DnBA | dibutyl adipate | 105-99-7 | TCI chemicals | DEHA-d8 | PA-ESI |
| DCaA | dicapryl adipate | 108-63-4 | Sigma-Aldrich | DEHA-d8 | PA-APCI |
| DiDeA | diisodecyl adipate | 27178-16-1 | AccuStandard | DEHA-d8 | PA-APCI |
| DEA | diethyl adipate | 141-28-6 | AccuStandard | DEHA-d8 | PA-ESI |
| DEHA | di(2-ethylhexyl) adipate | 103-23-1 | AccuStandard | DEHA-d8 | PA-ESI |
| DHeNoA | di(n-heptyl, n-nonyl) adipate | 68515-75-3 | AccuStandard | DEHA-d8 | PA-ESI |
| DMA | dimethyl adipate | 627-93-0 | AccuStandard | DEHA-d8 | PA-ESI |
| DiNA | di-iso-nonyl adipate | 33703-08-1 | TRC | DEHA-d8 | PA-APCI |
| Benzil | 1,2-diphenyl-1,2-ethanedione | 134-81-6 | TRC | DEHA-d8 | PA-ESI |
| BP | benzophenone | 119-61-9 | TRC | tert-paraben-d9 | PA-ESI |
| DETX | 2,4-diethyl-9H-thioxanthen-9-one | 82799-44-8 | TRC | DEHA-d8 | PA-ESI |
| DMAB | 4-(dimethylamino)benzophenone | 530-44-9 | TRC | DEHA-d8 | PA-ESI |
| EAB | ethyl-4-aminobenzoate | 94-09-7 | TRC | DEHA-d8 | PA-ESI |
| EAQ | 2-ethylanthraquinone | 84-51-5 | TRC | DEHA-d8 | PA-ESI |
| EDMAB | ethyl-4-dimethylaminobenzoate | 10287-53-3 | TRC | DEHA-d8 | PA-ESI |
| 2-ITX | 2-isopropylthioxanthone | 5495-84-1 | TRC | DEHA-d8 | PA-ESI |
| MBB | methyl-2-(benzoyl)benzoate | 606-28-0 | TRC | DEHA-d8 | PA-ESI |
| 4-MBP | 4-methylbenzophenone | 134-84-9 | TRC | DOP-d4 | PA-ESI |
| MEK | 4,4'-bis(diethylamino)benzophenone | 90-93-7 | TRC | DEHA-d8 | PA-ESI |
| MK | 4,4'-bis(dimethylamino)benzophenone | 90-94-8 | TRC | DEHA-d8 | PA-ESI |
| PBZ | 4-phenylbenzophenone | 2128-93-0 | TRC | DEHA-d8 | PA-ESI |
| PI-184 | 1-hydroxycyclohexyl phenyl ketone | 947-19-3 | TRC | DEHA-d8 | PA-ESI |
| PI-651 | 2,2-dimethoxy-2-phenylacetophenone | 24650-42-8 | TRC | DEHA-d8 | PA-ESI |
| BP-1 | 2,4-dihydroxybenzophenone | 131-56-6 | AccuStandard | 2,4-OH-BP-13C6 | PA-ESI |
| BP-2 | 2,2',4,4'-tetrahydroxybenzophenone | 131-55-5 | Sigma-Aldrich | 2,4-OH-BP-13C6 | PA-ESI |
| BP-3 | 2-hydroxy-4-methoxybenzophenone | 131-57-7 | AccuStandard | 2,4-OH-BP-13C6 | PA-ESI |
| BP-4 | 2-hydroxy-4-methoxybenzophenone-5-sulfonic acid hydrate | 4065-45-6 | TRC | 2,4-OH-BP-13C6 | PA-ESI |
| BP-6 | 2,2-dihydroxy-4,4-dimethoxybenzophenone | 131-54-4 | AccuStandard | 2,4-OH-BP-13C6 | PA-ESI |
| BP-8 | 2,2′-dihydroxy-4-methoxybenzophenone | 131-53-3 | TRC | 2,4-OH-BP-13C6 | PA-ESI |
| 4-OH-BP | 4-hydroxybenzophenone | 1137-42-4 | Sigma-Aldrich | 2,4-OH-BP-13C6 | PA-ESI |
| 2,3,4-OH-BP | 2,3,4-trihydroxybenzophenone | 1143-72-2 | Sigma-Aldrich | 2,4-OH-BP-13C6 | PA-ESI |
| BTH | benzothiazole | 95-16-9 | Sigma-Aldrich | BTH-d4 | PA-APCI |
| 2-Cl-BTH | 2-chlorobenzothiazole | 615-20-3 | TRC | BTH-d4 | PA-APCI |
| 2-Me-BTH | 2-methylbenzothiazole | 120-75-2 | TRC | BTH-d4 | PA-ESI |
| 2-Me-S-BTH | 2-(methylthio)benzothiazole | 615-22-5 | TCI chemicals | BTH-d4 | PA-ESI |
| 2-Mo-BTH | 2-(morpholinothio)-benzothiazole | 102-77-2 | TRC | BTH-d4 | PA-ESI |
| 2NH_2_-BTH | 2-aminobenzothiazole | 136-95-8 | TRC | BTH-d4 | PA-APCI |
| 2-OH-BTH | 2-hydroxybenzothiazole | 934-34-9 | TRC | BTH-d4 | PA-ESI |
| UV-P | 2-(2-hydroxy-5-methylphenyl) benzotriazole | 2440-22-4 | AccuStandard | 5-Me-BTR-d6 | PA-ESI |
| UV-PS | 2-(5-tert-butyl-2-hydroxyphenyl) benzotriazole | 3147-76-0 | AccuStandard | 5-Me-BTR-d6 | PA-ESI |
| UV-234 | 2-(2H-benzotriazol-2-yl)-4,6-bis(1-methyl-1-phenylethyl) phenol | 70321-86-7 | AccuStandard | 5-Me-BTR-d6 | PA-ESI |
| UV-320 | 2-(3,5-di-tert-butyl-2-hydroxyphenyl) 2H-benzotriazole | 3846-71-7 | AccuStandard | 5-Me-BTR-d6 | PA-ESI |
| UV-326 | 2-tert-butyl-6-(5-chloro-2H-benzotriazol-2-yl)-4-methylphenol | 3896-11-5 | AccuStandard | DEHA-d8 | PA-APCI |
| UV-327 | 2,4-di-tert-butyl-6-(5-chloro-2H-benzotriazol-2-yl) phenol | 3864-99-1 | AccuStandard | DEHA-d8 | PA-APCI |
| UV-328 | 2-(2H-benzotriazol-2-yl)-4,6-di-tert-pentylphenol | 25973-55-1 | AccuStandard | DEHA-d8 | PA-APCI |
| UV-350 | 2-(3-sec-butyl-5-tert-butyl-2-hydroxyphenyl) benzotriazole | 36437-37-3 | AccuStandard | 5-Me-BTR-d6 | PA-ESI |
| UV-360 | bisoctrizole | 103597-45-1 | TRC | DEHA-d8 | PA-APCI |
| 5-Cl-BTR | 5-chloro-1-hydrogenbenzotriazole | 94-97-3 | TRC | 5-Me-BTR-d6 | PA-ESI |
| 1-H-BTR | 1-hydrogen-benzotriazole | 95-14-7 | TRC | 1-H-BTR-d4 | PA-ESI |
| 1-OH-BTR | 1-hydroxybenzotriazole | 2592-95-2 | TCI chemicals | 1-H-BTR-d4 | PA-ESI |
| 4-Me-1-H-BTR | 4-methyl-1-hydrogenbenzotriazole | 29878-31-7 | TRC | 5-Me-BTR-d6 | PA-ESI |
| 5-Me-1-H-BTR | 5-methyl-1-hydrogenbenzotriazole | 136-85-6 | TRC | 5-Me-BTR-d6 | PA-ESI |
| 5,6-2Me-1-HBTR | 5,6-dimethyl-1-hydrogenbenzotriazole | 4184-79-6 | TCI chemicals | DEHA-d8 | PA-ESI |
| BPA | bisphenol A | 80-05-7 | AccuStandard | BPA-d6 | PA-ESI |
| BPAF | bisphenol AF | 1478-61-1 | AccuStandard | BPS-C12 | PA-ESI |
| BPAP | bisphenol AP | 1571-75-1 | AccuStandard | BPS-C12 | PA-ESI |
| BPB | bisphenol B | 77-40-7 | AccuStandard | BPS-C12 | PA-ESI |
| BPBP | bisphenol BP | 1844-01-5 | AccuStandard | BPS-C12 | PA-ESI |
| BPC | bisphenol C | 79-97-0 | AccuStandard | BPS-C12 | PA-ESI |
| BPE | bisphenol E | 2081-08-5 | AccuStandard | BPS-C12 | PA-ESI |
| BPF | bisphenol F | 620-92-8 | AccuStandard | BPS-C12 | PA-ESI |
| BPG | bisphenol G | 127-54-8 | AccuStandard | BPS-C12 | PA-ESI |
| BPM | bisphenol M | 13595-25-0 | AccuStandard | BPS-C12 | PA-ESI |
| BPP | bisphenol P | 2167-51-3 | AccuStandard | BPS-C12 | PA-ESI |
| BPPH | bisphenol PH | 24038-68-4 | AccuStandard | BPS-C12 | PA-ESI |
| BPS | bisphenol S | 80-09-1 | AccuStandard | BPS-C12 | PA-ESI |
| BPZ | bisphenol Z | 843-55-0 | AccuStandard | BPS-C12 | PA-ESI |
| ATBC | acetyl tri-n-butyl citrate | 77-90-7 | AccuStandard | DEHA-d8 | PA-ESI |
| ATEC | acetyl triethyl citrate | 77-89-4 | AccuStandard | DEHA-d8 | PA-ESI |
| BTHC | n-butyryl tri-n-hexyl citrate | 82469-79-2 | AccuStandard | DEHA-d8 | PA-ESI |
| TBC | tributyl citrate | 77-94-1 | AccuStandard | DEHA-d8 | PA-ESI |
| TEC | triethyl citrate | 77-93-0 | AccuStandard | DEHA-d8 | PA-ESI |
| TPIB | 2,2,4-trimethyl-1,3-pentanediol-monoisobutyrate | 25265-77-4 | AccuStandard | DEHA-d8 | PA-ESI |
| TXIB | 2,2,4-trimethyl-1,3-pentanediol-diisobutyrate | 6846-50-0 | AccuStandard | DEHA-d8 | PA-ESI |
| BHA | 3-tert-butyl-4-hydroxyanisole | 121-00-6 | AccuStandard | BHT-d21 | PA-APCI |
| BHT | 2,6-di-tert-butyl-4-hydroxytoluene | 128-37-0 | AccuStandard | BHT-d21 | PA-APCI |
| BHT-CHO | 3,5-di-tert-butyl-4-hydroxybenzaldehyde | 1620-98-0 | AccuStandard | BHT-d21 | PA-APCI |
| BHT-COOH | 3,5-di-tert-butyl-4-hydroxybenzoic acid | 1421-49-4 | TCI chemicals | BHT-d21 | PA-APCI |
| BHT-OH | 2,6-di-tert-butyl-4-(hydroxymethyl)phenol | 88-26-6 | AccuStandard | BHT-d21 | PA-APCI |
| BHT-Q | 2,6-di-tert-butyl-1,4-benzoquinone | 719-22-2 | TRC | BHT-d21 | PA-APCI |
| BHT-quinol | 2,6-di-tert-butyl-4-hydroxy-4-methyl-2,5-cyclohexadienone | 10396-80-2 | TRC | BHT-d21 | PA-APCI |
| BMDM | 4-tert-butyl-4′-methoxydibenzoylmethane | 70356-09-1 | AccuStandard | 5-Me-BTR-d6 | PA-ESI |
| EHS | 2-ethylhexyl salicylate | 118-60-5 | AccuStandard | DEHA-d8 | PA-APCI |
| HMS | 3,3,5-trimethylcyclohexyl salicylate | 118-56-9 | AccuStandard | DEHA-d8 | PA-APCI |
| IAMC | isoamyl 4-methoxycinnamate | 71617-10-2 | TCI chemicals | 5-Me-BTR-d6 | PA-ESI |
| 4-MBC | 4-methylbenzylidene camphor | 36861-47-9 | AccuStandard | DEHA-d8 | PA-APCI |
| ODPABA | octyl dimethyl-p-aminobenzoic acid | 21245-02-3 | AccuStandard | DEHA-d8 | PA-ESI |
| OC | 2-ethylhexyl 2-cyano-3,3-diphenyl-2-propenoate | 6197-30-4 | DR.Ehrenstorfer | DOP-d4 | PA-ESI |
| OMC | ethylhexyl methoxycinnamate | 5466-77-3 | AccuStandard | DEHA-d8 | PA-ESI |
| Cyanox 1212 | dodecyl octadecyl 3,3'-sulfanediyldipropanoate | 13103-52-1 | AccuStandard | DOP-d4 | PA-ESI |
| Cyanox 1790 | tris(4-tert-butyl-3-hydroxy-2,6-dimethylbenzyl) isocyanurate | 40601-76-1 | AccuStandard | DOP-d4 | PA-ESI |
| Ethanox 703 | 2,6-di-tert-butyl-4-(dimethylamino-methyl)phenol | 88-27-7 | AccuStandard | DOP-d4 | PA-ESI |
| Irganox 1035 | thiodiethylene bis(3-(3,5-di-tert-butyl-4-hydroxyphenyl)propionate) | 41484-35-9 | AccuStandard | DOP-d4 | PA-ESI |
| Irganox 1081 | 2,2'-thiobis(6-tert-butyl-p-cresol) | 90-66-4 | AccuStandard | tert-paraben-d9 | PA-ESI |
| Irganox 1098 | N,N'-1,6-hexanediylbis{3-[4-hydroxy-3,5-bis(2-methyl-2-propanyl)phenyl]propanamide} | 23128-74-7 | AccuStandard | DOP-d4 | PA-ESI |
| Irganox 245 | triethylene glycol bis(3-tert-butyl-4-hydroxy-5-methylphenyl)propionate | 36443-68-2 | AccuStandard | DOP-d4 | PA-ESI |
| Irganox 259 | benzenepropanoic acid, 3,5-bis(1,1-dimethylethyl)-4-hydroxy-, 1,6-hexanediyl ester | 35074-77-2 | AccuStandard | DOP-d4 | PA-ESI |
| Irganox 330 | 1,3,5-trimethyl-2,4,6-tris(3,5-di-tert-butyl-4-hydroxybenzyl)benzene | 1709-70-2 | AccuStandard | DOP-d4 | PA-ESI |
| Irganox 445 | bis[4-(2-phenyl-2-propyl)phenyl]amine | 10081-67-1 | AccuStandard | DOP-d4 | PA-ESI |
| Irganox 565 | 4-[[4,6-bis(octylsulfanyl)-1,3,5-triazin-2-yl]amino]-2,6-ditert-butylphenol | 991-84-4 | AccuStandard | DOP-d4 | PA-ESI |
| Irganox 697 | (1,2-dioxo-1,2-ethanediyl)bis(imino-2,1-ethanediyl) bis{3-[4-hydroxy-3,5-bis(2-methyl-2-propanyl)phenyl]propanoate} | 70331-94-1 | AccuStandard | DOP-d4 | PA-ESI |
| AO1024 | 1,2-bis(3,5-di-tert-butyl-4-hydroxyhydrocinnamoyl)hydrazine | 32687-78-8 | AccuStandard | tert-paraben-d9 | PA-ESI |
| AO1222 | diethyl-3,5-di-tert-butyl-4-hydroxybenzyl phosphonate | 976-56-7 | AccuStandard | tert-paraben-d9 | PA-ESI |
| AO168 | tris(2,4-ditert-butylphenyl) phosphite | 31570-04-4 | AccuStandard | DOP-d4 | PA-APCI |
| AO2246 | 2,2'-methylenebis(6-tert-butyl-4-methylphenol) | 119-47-1 | AccuStandard | tert-paraben-d9 | PA-ESI |
| AO22E46 | 2,2'-ethylidene-bis(4,6-di-tert-butylphenol) | 35958-30-6 | AccuStandard | DOP-d4 | PA-ESI |
| AO4426 | 4,4'-methylenebis(2,6-di-tert-butylphenol) | 118-82-1 | AccuStandard | DEHA-d8 | PA-APCI |
| AO44B25 | 4,4'-butylidenebis(6-tert-butyl-m-cresol) | 85-60-9 | AccuStandard | tert-paraben-d9 | PA-ESI |
| BBOT | 2,2'-(2,5-thiophenediyl)-bis (5-tert-butylbenzoxazole) | 7128-64-5 | AccuStandard | DOP-d4 | PA-ESI |
| DBHA | dibenzylhydroxylamine | 621-07-8 | AccuStandard | DOP-d4 | PA-ESI |
| 2,4DtBP | 2,4-di-tert-butylphenol | 96-76-4 | AccuStandard | tert-paraben-d9 | PA-APCI |
| DET | N,N'-diethylthiourea | 105-55-5 | AccuStandard | DOP-d4 | PA-ESI |
| DPPD | N,N'-diphenyl-1,4-benzenediamine | 74-31-7 | AccuStandard | DOP-d4 | PA-ESI |
| 3,5-DTBH | 11-methyldodecyl 3-[4-hydroxy-3,5-bis(2-methyl-2-propanyl)phenyl]propanoate | 847488-62-4 | AccuStandard | DOP-d4 | PA-ESI |
| DTBNP | 2,6-di-tert-butyl-4-nonylphenol | 4306-88-1 | AccuStandard | DEHA-d8 | PA-APCI |
| DTG | 1,3-di-o-tolylguanidine | 97-39-2 | AccuStandard | DOP-d4 | PA-ESI |
| DPG | 1,3-diphenylguanidine | 102-06-7 | AccuStandard | DOP-d4 | PA-ESI |
| MBEBP | 2,2'-methylenebis(4-ethyl-6-tert-butylphenol) | 88-24-4 | AccuStandard | tert-paraben-d9 | PA-ESI |
| MMBI | methyl-2-mercaptobenzimidazole | 53988-10-6 | AccuStandard | tert-paraben-d9 | PA-ESI |
| NPN | N-phenyl-1-naphthylamine | 90-30-2 | AccuStandard | DOP-d4 | PA-ESI |
| 4-tOP | 4-(1,1,3,3-tetra-methylbutyl)phenol | 140-66-9 | TCI chemicals | tert-paraben-d9 | PA-APCI |
| TBBC | 4,4'-thiobis(6-tert-butyl-m-cresol) | 96-69-5 | AccuStandard | tert-paraben-d9 | PA-ESI |
| **Surrogate and internal standards** | | | | | |
| **Commercial name or abbreviation** | **Full Name** | **CAS number** | **Supplier** | **Type** | **Quantification methods** |
| DBP-d4 | di-n-butyl phthalate-d4 | 93952-11-5 | AccuStandard | Surrogate | PA-ESI |
| DBzP-d4 | dibenzylphthalate-d4 | 1015854-62-2 | AccuStandard | Surrogate | PA-ESI |
| DEP-d4 | diethyl phthalate-3,4,5,6-d4 | 93952-12-6 | AccuStandard | Surrogate | PA-ESI |
| DEHP-d4 | bis(2-ethylhexyl) phthalate-3,4,5,6-d4 | 93951-87-2 | AccuStandard | Surrogate | GC |
| DHxP-d4 | di-n-hexyl phthalate-3,4,5,6-d4 | 1015854-55-3 | AccuStandard | Surrogate | PA-ESI |
| DMP-d4 | dimethyl phthalate-3,4,5,6-d4 | 93951-89-4 | AccuStandard | Surrogate | PA-ESI |
| DOP-d4 | di-n-octyl phthalate-3,4,5,6-d4 | 93952-13-7 | AccuStandard | Surrogate | PA-ESI, PA-APCI |
| DPeP-d4 | di-n-pentyl phthalate-3,4,5,6-d4 | 358730-89-9 | AccuStandard | Surrogate | PA-ESI |
| DPrP-d4 | di-n-propyl phthalate-3,4,5,6-d4 | 358731-29-0 | AccuStandard | Surrogate | PA-ESI |
| MBP-d4 | mono-n-butyl phthalate-d4 | 478954-81-3 | CDN Isotopes | Surrogate | PA-ESI |
| MBzP-d4 | mono-benzyl phthalate-d4 | 478954-83-5 | CDN Isotopes | Surrogate | PA-ESI |
| MEP-d4 | monoethyl phthalate-d4 | 1219806-03-7 | TRC | Surrogate | PA-ESI |
| MEHP-d4 | rac mono(ethylhexyl) phthalate-d4 | 1276197-22-8 | TRC | Surrogate | PA-ESI |
| DEHA-d8 | bis(2-ethylhexyl) adipate-d8 | 1214718-98-5 | TRC | Surrogate | PA-ESI, PA-APCI |
| DEHT-d4 | bis(2-ethylhexyl) terephthalate-d4 | NA^c^ | TRC | Surrogate | GC |
| d-TBP | tri-n-butyl phosphate-d27 | 61196-26-7 | Wellington | Surrogate | PA-ESI |
| M6TBOEP | tris(2-butoxy-[13C2]-ethyl) phosphate | NA | Wellington | Surrogate | PA-ESI |
| d-TCEP | tris(2-chloroethyl) phosphate-d12 | 1276500-47-0 | Wellington | Surrogate | PA-ESI |
| d-TDCPP | tris(1,3-dichloro-2-propyl) phosphate-d15 | 1447569-77-8 | Wellington | Surrogate | PA-ESI |
| d-TEP | triethyl phosphate-d15 | 135942-11-9 | Wellington | Surrogate | PA-ESI |
| d-TPP | triphenyl phosphate-d15 | 1173020-30-8 | Wellington | Surrogate | PA-ESI |
| tert-paraben-d9 | tert-paraben-d9 | 1216904-65-2 | TRC | Surrogate | PA-ESI, PA-APCI |
| 2,4-OH-BP-13C6 | 2,4-dihydroxybenzophenone-13C6 | 2731164-01-3 | TRC | Surrogate | PA-ESI, PA-APCI |
| BTH-d4 | benzothiazole-d4 | 194423-51-3 | TRC | Surrogate | PA-ESI |
| 1-H-BTR-d4 | 1H-benzotriazole-(ring- d4) | 1185072-03-0 | TRC | Surrogate | PA-ESI |
| 5-Me-BTR-d6 | 5-methyl-benzotriazole-d6 | 1246820-65-4 | TRC | Surrogate | PA-ESI |
| BPA-d6 | bisphenol A-d6 | 86588-58-1 | TRC | Surrogate | PA-ESI |
| BPS-C12 | bisphenol S-13C12 | 1991267-29-8 | TRC | Surrogate | PA-ESI |
| BHT-d21 | 2,6-di(tert-butyl)-4-methyl-phenol-d21 | 64502-99-4 | DR.Ehrenstorfer | Surrogate | PA-ESI, PA-APCI |
| DCDE | decachlorodiphenyl ether | 31710-30-2 | AccuStanard | Internal | GC |
| BPA-d16 | bisphenol A-d16 | 96210-87-6 | Sigma-Aldrich | Internal | PA-ESI, PA-APCI |
| Coumaphos-d10 | coumaphos-d10 | 287397-86-8 | TRC | Internal | PA-ESI, PA-APCI |
| **Reagent** | | | | | |
| **Reagent** |  | **CAS** | **Supplier** | | **Catalogue number** |
| LC-MS grade acetonitrile | | 75-05-8 | Supelco | | K53971030 206 |
| LC-MS grade dichloromethane | | 75-09-2 | Oceanpak | | 16101 |
| LC-MS grade hexane | | 110-54-3 | Fisher Scientific | | H303-4 |
| LC-MS grade methanol | | 67-56-1 | Fisher Scientific | | 219089 |
| LC-MS grade water | | 7732-18-5 | Fisher Scientific | | 219205 |

^a^ Details of instrumental methods

1. **PA-ESI**: The instrument system consists of an ultra-performance liquid chromatography coupled to a 5500 triple quadrupole mass spectrometer (AB Sciex, Toronto, Canada). LC separation was achieved on a Luna^®^ Omega 3 μm PS C18 (2) 100Å column (100 mm × 3.0 mm; 3 μm particle size; Phenomenex, Torrance, CA, U.S.) with a flow rate of 300 μL/min. The mobile phase is water spiked with 0.2 mM ammonium acetate (solvent A) and methanol (solvent B). The gradient was initiated with 10% solvent B and equilibrated for 2 min, and continued with a linear increase to 70% B in 4 min, and followed by a linear increase to 99% B in 11 min and held for 5 min. After which the system was returned to 10% B and was re-equilibrated for 3 min. The MS was equipped with a TurboIonSpray® electrospray ionization (ESI) probe and conducted in the positive multiple reaction monitoring (MRM) mode.
2. **PA-APCI**: A UPLC-MS/MS was used (Triple Quad 5500, AB Sciex, Toronto, Canada). LC separation was achieved by using an ACQUITY UPLC BEH Shield RP18 130Å column (100 mm × 2.1 mm; 1.7 μm particle size; Waters, Milford, U.S.) with a flow rate of 300 μL/min. The mobile phase consisted of water (solvent A) and methanol (solvent B). The gradient was programmed as: 0-1.5 min, 55% solvent B; followed by a linear increase to 100% B in 8.5 min (held for 5 min), and then a change to 55% B in 0.5 min and held for 5 min. The MS was equipped with an atmospheric pressure chemical ionization (APCI) source and conducted in both positive and negative MRM mode.
3. **GC**: A gas chromatograph interfaced with a mass spectrometer (GC-MS) in electron impact mode (7890B-5977A, Agilent, USA) was used. Separation was achieved on a 30-m HP-5MS column (0.25 mm i.d., 0.25 μm, J&W Scientific, Agilent, U.S.). The GC injector was operated in spitless mode and set as 260 °C. The initial oven temperature was set as 80 °C and held for 3 min, and then ramped to 150 °C at 10 °C/min, and increased to 300 °C at 5 °C/min (held for 10 min). The heated transfer line temperature and quadrupole temperature were set as 280 °C and 150 °C, respectively.

^b^ Toronto Research Chemical; ^c^NA: not available.

**Table S4.** Major chemical-dependent parameters for instrumental determination of plastic additives, including the multiple-reaction-monitoring (MRM) ion transitions, declustering potential (DP), and collision energy (CE).

| **Commercial name or abbreviation** | **Quantitative ion** | | | **Qualitative ion** | | |
| --- | --- | --- | --- | --- | --- | --- |
|  | **Ion transition** | **DP** | **CE** | **Ion transition** | **DP** | **CE** |
| **Target plastic additives** | | | | | | |
| BBzPh | 313.1 → 91.1 | 11 | 19 | 313.1 → 149.2 | 11 | 19 |
| iBCHPh | 305.2 → 149.1 | 21 | 25 | 305.2 → 223.1 | 21 | 25 |
| BMPP | 335.1 → 149 | 70 | 40 | 335.1 → 84.8 | 80 | 40 |
| DAlP | 247.1 → 189.3 | 60 | 12 | 247.1 → 149.0 | 60 | 12 |
| DAmP | 307.0 → 149.1 | 21 | 21 | 307.0 → 65.1 | 21 | 75 |
| DiBP | 279.2 → 149.1 | 26 | 21 | 279.2 → 65.1 | 26 | 21 |
| DBP | 208.9 → 78.8 | -100 | 21 | 208.9 → 153.2 | -90 | 13 |
| DBzP | 347.1 → 91.0 | 100 | 20 | 347.1 → 181.2 | 10 | 10 |
| DEP | 223.0 → 149.0 | 26 | -32 | 223.0 → 177.0 | 26 | -32 |
| DEHP | 391.4 → 149.1 |  | 23 | 391.4 → 71.2 |  | 23 |
| DiHeP | 363.2 → 99.2 | 31 | 23 | 363.2 → 149.2 | 31 | 17 |
| DiHxP | 335.1 → 85.2 | 26 | 21 | 335.1 → 149 | 26 | 21 |
| DHxP | 335.1 → 85.3 | 21 | 19 | 335.1 → 149.1 | 21 | 17 |
| DMiP | 195.1 → 105.0 | 70 | 20 | 195.1 → 91.1 | 70 | 20 |
| DMP | 195.1 → 163.1 | 31 | -36 | 195.1 → 77.0 | 31 | -36 |
| DiNP | 419.3 → 85.1 | 26 | 27 | 419.3 → 220.8 | 26 | 23 |
| DNP | 419.3 → 149.1 | 31 | 21 | 419.3 → 71.2 | 31 | 67 |
| DiPeP | 307.2 → 71.2 | 26 | 21 | 307.2 → 149.1 | 26 | 21 |
| DPHiP | 319 → 224.9 | 100 | 20 | 319 → 77.1 | 20 | 100 |
| DPHP | 319.0 → 224.9 | 70 | 20 | 319.1 → 77.1 | 70 | 70 |
| DiPrP | 251.2 → 149.1 | 21 | 21 | 251.2 → 65.1 | 21 | 67 |
| DPrP | 251.2 → 120.9 | 80 | 40 | 251.2 → 42.9 | 20 | 40 |
| DUP | 475.1 → 149 | 36 | 23 | 475.1 → 120.9 | 36 | 71 |
| MiBP | 221.0 → 77.0 | -20 | -24 | 221.0 → 134.0 | -20 | -20 |
| MBP | 221.0 → 77.0 | -20 | -24 | 221.0 → 134.0 | -20 | -20 |
| MBzP | 255.1 → 77.0 | -30 | -30 | 255.1 → 107.0 | -30 | -20 |
| MCHP | 247.0 → 97.0 | -35 | -30 | 247.0 → 77.0 | -35 | -24 |
| MEP | 193.0 → 77.0 | -20 | -24 | 193.0 → 121 | -20 | -18 |
| MEHP | 277.0 → 127.1 | -30 | -22 | 277.0 → 134.1 | -25 | -36 |
| MECPP | 306.9 → 159.1 | -60 | -20 | 306.9 → 113.1 | -60 | -35 |
| MEHHP | 293.1 → 121.1 | -30 | -21 | 293.1 → 144.7 | -45 | -21 |
| MEOHP | 291.0 → 121.1 | -35 | -24 | 291.0 → 143.1 | -35 | -26 |
| MHeP | 263.0 → 113.1 | -25 | -20 | 263.0 → 77.0 | -25 | -34 |
| MHxP | 249.0 → 77.0 | -25 | -30 | 249.0 → 99.1 | -25 | -22 |
| MMP | 179 → 77.1 | -25 | -24 | 179 → 107 | -25 | -14 |
| MiNP | 291 → 141.1 | -35 | -26 | 291 → 77 | -35 | -36 |
| MOP | 277 → 77.1 | -30 | -24 | 277 → 127.1 | -30 | -34 |
| MPeP | 235.0 → 77.0 | -25 | -26 | 235.0 → 85.0 | -25 | -20 |
| MiPrP | 207.1 → 77.0 | -25 | -22 | 207.1 → 120.9 | -25 | -20 |
| BARO | 397.5 → 337.1 | 58 | 9 | 397.5 → 263.2 | 56 | 16 |
| BRO | 355.5 → 337.2 | 62 | 7 | 355.5 → 263.2 | 44 | 13 |
| BO | 339.5 → 265.2 | 62 | 15 | 339.5 → 247.2 | 70 | 18 |
| DBF | 229.3 → 117.1 | 59 | 16 | 229.3 → 57.0 | 71 | 20 |
| DBS | 315.4 → 241.2 | 30 | 19 | 315.4 → 185.2 | 31 | 12 |
| DEHM | 341.4 → 99.1 | 37 | 13 | 341.4 → 117.1 | 56 | 37 |
| DES | 175.2 → 129.1 | 28 | 11 | 175.2 → 101.1 | 30 | 17 |
| DMS | 231.2 → 199.3 | 25 | 11 | 231.2 → 139.3 | 25 | 20 |
| EHSe | 427.5 → 185.1 | 62 | 18 | 427.5 → 315.3 | 27 | 11 |
| GMO | 357.4 → 265.4 | 63 | 13 | 357.4 → 247.3 | 50 | 17 |
| MO | 297.4 → 247.1 | 49 | 15 | 297.4 → 265.3 | 53 | 11 |
| MOA | 355.4 → 295.2 | 62 | 9 | 355.4 → 263.2 | 80 | 13 |
| IPM | 271.4 → 229.2 | 11 | 10 | 271.4 → 103.1 | 36 | 20 |
| IPP | 316.5 → 257.2 | 48 | 15 | 316.5 → 299.4 | 47 | 7 |
| PO | 325.4 → 265.1 | 63 | 15 | 325.4 → 247.3 | 41 | 17 |
| THFO | 367.4 → 85.1 | 10 | 16 | 367.4 → 265.3 | 49 | 16 |
| DEHT | 391.4 → 149.1 |  | 25 | 391.4 → 71.2 |  | 25 |
| DEGDB | 315.3 → 149.1 | 39 | 41 | 315.3 → 105.1 | 43 | 12 |
| DPGDB | 343.3 → 163.1 | 47 | 37 | 343.3 → 105.1 | 77 | 37 |
| DINCH | 425.4 → 71.1 | 80 | 22 | 425.4 → 155.2 | 80 | 22 |
| DiDEAZ | 469.0 → 170.9 | 21 | 24 | 469.0 → 71.2 | 21 | 25 |
| DHAZ | 357.5 → 255.2 | 77 | 20 | 357.5 → 171.1 | 54 | 14 |
| DMAZ | 217.1 → 185.2 | 61 | 10 | 217.1 → 97.1 | 54 | 20 |
| DiOAZ | 413.5 → 283.2 | 31 | 13 | 413.5 → 171.1 | 18 | 18 |
| TCaT | 574.1 → 193 | 36 | 55 | 574.1 → 323 | 36 | 21 |
| THTM | 463.4 → 277.2 | 37 | 17 | 463.4 → 361.3 | 52 | 11 |
| TiNTM | 589.4 → 127.2 | 51 | 18 | 589.4 → 319.1 | 19 | 29 |
| TOTM | 574.2 → 193.1 | 16 | 60 | 574.2 → 305.2 | 16 | 30 |
| TiTDM | 631.1 → 268.9 | 46 | 37 | 631.1 → 72.8 | 46 | 41 |
| TBP | 267.1 → 98.9 | 40 | 23 | 267.1 → 80.9 | 40 | 70 |
| TBOEP | 399.1 → 199.1 | 30 | 20 | 399.1 → 101.0 | 30 | 20 |
| TCEP | 284.9 → 99.0 | 100 | 28 | 284.9 → 62.9 | 100 | 20 |
| TCIPP | 329.0 → 99.0 | 70 | 33 | 329.0 → 81.0 | 70 | 89 |
| TDBPP | 698.4 → 98.9 | 80 | 85 | 698.4 → 118.9 | 80 | 145 |
| TDCPP | 430.9 → 99.0 | 60 | 34 | 430.9 → 81.0 | 60 | 102 |
| TEP | 183.1 → 99.0 | 50 | 24 | 183.1 → 81.0 | 50 | 52 |
| TEHP | 435.3 → 99.0 | 50 | 15 | 435.3 → 81.0 | 50 | 60 |
| TPrP | 225.1 → 98.9 | 60 | 28 | 225.1 → 80.9 | 60 | 61 |
| V6 | 582.9 → 65.1 | 120 | 72 | 582.9 → 99.1 | 120 | 104 |
| BPADP | 693.0 → 367.2 | 15 | 48 | 693.0 → 178.2 | 15 | 78 |
| CDP | 340.9 → 91.1 | 190 | 51 | 340.9 → 151.9 | 190 | 51 |
| DDPP | 391.0 → 251.1 | 40 | 21 | 391.0 → 77.1 | 40 | 76 |
| EHDPP | 363.2 → 250.9 | 60 | 39 | 363.2 → 76.9 | 60 | 22 |
| RDP | 575.0 → 77.1 | 200 | 124 | 575.0 → 152.2 | 200 | 110 |
| TCrP | 369.1 → 165.0 | 200 | 62 | 369.1 → 91.0 | 200 | 62 |
| T34DMPP | 411.2 → 178.8 | 187 | 51 | 411.2 → 194.2 | 201 | 41 |
| T35DMPP | 411.1 → 179.1 | 190 | 51 | 411.1 → 194.0 | 190 | 41 |
| TPHP | 327.1 → 77.1 | 180 | 64 | 327.1 → 152.1 | 180 | 52 |
| B2tBPPP | 439.3 → 383.2 | 186 | 24 | 439.3 → 327.0 | 266 | 47 |
| B4tBPPP | 439.3 → 383.2 | 120 | 24 | 439.3 → 327.0 | 120 | 47 |
| 2tBPDPP | 383.1 → 327.0 | 166 | 42 | 383.1 → 251.0 | 161 | 47 |
| 4tBPDPP | 383.1 → 327.0 | 143 | 42 | 383.2 → 233.0 | 175 | 47 |
| 24DIPPDPP | 411.2 → 369.0 | 166 | 23 | 411.2 → 327.0 | 181 | 37 |
| B24DIPPPP | 495.3 → 411.0 | 185 | 35 | 495.3 → 369.0 | 200 | 42 |
| B2IPPPP | 411.3 → 369.0 | 171 | 23 | 411.3 → 327.0 | 173 | 37 |
| B4IPPPP | 411.3 → 369.1 | 190 | 23 | 411.3 → 327.0 | 178 | 37 |
| 2IPPDPP | 369.2 → 327.0 | 165 | 27 | 369.2 → 233.0 | 162 | 37 |
| 4IPPDPP | 369.2 → 327.0 | 181 | 29 | 369.2 → 233.0 | 153 | 39 |
| T4tBPP | 495.3 → 439.0 | 195 | 38 | 495.3 → 383.0 | 210 | 32 |
| T2IPPP | 452.9 → 326.9 | 180 | 41 | 452.9 → 91.0 | 180 | 38 |
| T3IPPP | 453.2 → 411.3 | 210 | 41 | 453.2 → 369.3 | 223 | 38 |
| T4IPPP | 453.2 → 411.3 | 176 | 41 | 453.2 → 369.3 | 176 | 38 |
| DiBA | 259.1 → 129.1 | 26 | 13 | 259.1 → 111.0 | 26 | 21 |
| DnBA | 259.1 → 185.2 | 26 | 13 | 259.1 → 111.0 | 26 | 21 |
| DCaA | 371.3 → 147.0 | 80 | 17 | 371.3 → 129.0 | 110 | 41 |
| DiDeA | 427.1 → 129.1 | 21 | 25 | 427.1 → 85.1 | 21 | 29 |
| DEA | 203.3 → 157.1 | 43 | 9 | 203.3 → 111.0 | 33 | 18 |
| DEHA | 371.1 → 129.1 | 21 | 19 | 371.1 → 101.1 | 21 | 37 |
| DHeNoA | 371.1 → 129.1 | 51 | 21 | 371.1 → 101.1 | 51 | 29 |
| DMA | 175.3 → 143.1 | 62 | 15 | 175.3 → 111.1 | 51 | 8 |
| DiNA | 399.4 → 129.1 | 60 | 26 | 399.4 → 111.1 | 103 | 26 |
| Benzil | 211.2 → 105.0 | 115 | 15 | 211.2 → 77.1 | 115 | 48 |
| BP | 183.1 → 77.1 | 110 | 22 | 183.1 → 105.1 | 110 | 44 |
| DETX | 269.3 → 241.1 | 190 | 30 | 269.3 → 213.0 | 190 | 39 |
| DMAB | 226.3 →105.1 | 160 | 27 | 226.3 → 77.1 | 160 | 55 |
| EAB | 166.2 → 138.1 | 90 | 15 | 166.2 → 127.1 | 90 | 38 |
| EAQ | 237.0 → 209.2 | 170 | 30 | 237.0 → 153.0 | 170 | 38 |
| EDMAB | 194.3 → 151.2 | 150 | 32 | 194.3 → 166.3 | 150 | 23 |
| 2-ITX | 255.3 → 213.1 | 190 | 30 | 255.3 → 184.0 | 190 | 53 |
| MBB | 241.2 → 209.1 | 100 | 20 | 241.2 → 152.2 | 100 | 50 |
| 4-MBP | 197.2 → 105.1 | 150 | 25 | 197.2 → 119.0 | 150 | 21 |
| MEK | 325.4 → 176.1 | 100 | 38 | 325.4 → 133.1 | 100 | 62 |
| MK | 269.3 → 148.1 | 150 | 34 | 269.3 → 120.1 | 150 | 43 |
| PBZ | 259.2 → 105.1 | 150 | 26 | 259.2 → 77.2 | 150 | 55 |
| PI-184 | 205.2 → 105.1 | 90 | 18 | 205.2 → 187.1 | 90 | 9 |
| PI-651 | 225.2 → 197.1 | 140 | 21 | 225.2 → 105.1 | 140 | 30 |
| BP-1 | 213.0 → 91.0 | -130 | -35 | 213.0 → 135.0 | -130 | -25 |
| BP-2 | 245.0 → 135.0 | -114 | -25 | 245.0 → 109.0 | -109 | -28 |
| BP-3 | 227.0 → 199.0 | -160 | -50 | 227.0 → 101.0 | -147 | -25 |
| BP-4 | 307.0 → 227.0 | -92 | -36 | 307.0 → 211.0 | -88 | -46 |
| BP-6 | 259.0 → 109.0 | -54 | -23 | 259.0 → 135.0 | -52 | -26 |
| BP-8 | 243.0 → 93.0 | -72 | -27 | 243.0 → 123.0 | -82 | -22 |
| 4-OH-BP | 197.0 → 92.0 | -130 | -41 | 197.0 → 120.0 | -128 | -32 |
| 2,3,4-OH-BP | 229.0 → 123.0 | -80 | -36 | 229.0 → 107.0 | -82 | -33 |
| BTH | 136.0 → 109.2 | 60 | 22 | 136.0 → 65.2 | 60 | 22 |
| 2-Cl-BTH | 170.1 → 93.0 | 48 | 39 | 170.1 → 65.0 | 68 | 53 |
| 2-Me-BTH | 150.0 → 108.8 | 109 | 31 |  |  |  |
| 2-Me-S-BTH | 182.0 → 109.1 | 100 | 50 | 182.0 → 65.1 | 100 | 70 |
| 2-Mo-BTH | 253.2 → 86.2 | 36 | 25 | 253.2 → 55.8 | 36 | 31 |
| 2NH_2_-BTH | 151.0 → 124.1 | 60 | 22 | 151.0 → 109.1 | 60 | 22 |
| 2-OH-BTH | 152.1 → 124.1 | 70 | 30 | 152.1 → 92.1 | 90 | 30 |
| UV-P | 226.0 → 120.2 | 41 | 27 | 226.0 → 77.2 | 41 | 51 |
| UV-PS | 268.2 → 212.2 | 41 | 23 | 268.2 → 65.2 | 41 | 73 |
| UV-234 | 448.1 → 91.0 | 22 | 83 | 448.1 → 370.3 | 22 | 30 |
| UV-320 | 324.2 → 57.0 | 80 | 40 | 324.2 → 212.1 | 90 | 23 |
| UV-326 | 316.1 → 260.2 | 30 | 30 | 316.1 → 107.2 | 30 | 40 |
| UV-327 | 358.1 → 301.9 | 20 | 31 | 358.1 → 246.0 | 20 | 45 |
| UV-328 | 352.1 → 282.2 | 21 | 25 | 352.1 → 70.9 | 20 | 27 |
| UV-350 | 324.2 → 212.1 | 90 | 40 | 324.2 → 268.2 | 40 | 23 |
| UV-360 | 659.3 → 238.0 | 11 | 31 | 659.3 → 207.0 | 19 | 35 |
| 5-Cl-BTR | 154.0 → 99.3 | 36 | 30 |  |  |  |
| 1-H-BTR | 120.1 → 65.1 | 70 | 30 | 120.1 → 92.2 | 70 | 20 |
| 1-OH-BTR | 136.0 → 64.1 | 36 | 30 |  |  |  |
| 4-Me-1-H-BTR | 134.2 → 79.2 | 36 | 30 |  |  |  |
| 5-Me-1-H-BTR | 134.1 → 77.1 | 36 | 35 | 134.1 → 79.0 | 36 | 35 |
| 5,6-2Me-1-HBTR | 148.0 → 93.3 | 36 | 30 |  |  |  |
| BPA | 227.2 → 211.0 | -17 | -36 | 227.2 → 133.0 | -18 | -26 |
| BPAF | 335.1 → 264.9 | -30 | -30 | 335.1 → 69.1 | -30 | -69 |
| BPAP | 289.1 → 211.0 | -70 | -48 | 289.1 → 195.0 | -70 | -31 |
| BPB | 240.9 → 211.0 | -60 | -42 |  |  |  |
| BPBP | 351.1 → 272.8 | -20 | -35 | 351.1 → 258.1 | -20 | -42 |
| BPC | 255.1 → 146.7 | -80 | -40 | 255.1 → 107.2 | -80 | -60 |
| BPE | 213.1 → 118.8 | -30 | -32 | 213.1 → 92.7 | -30 | -34 |
| BPF | 199.2 → 105.0 | -12 | -28 | 199.2 → 77.0 | -29 | -28 |
| BPG | 311.3 → 174.8 | -22 | -47 |  |  |  |
| BPM | 345.1 → 251.1 | -30 | -43 | 345.1 → 132.7 | -30 | -52 |
| BPP | 345.2 → 132.9 | -30 | -52 | 345.2 → 315.4 | -28 | -46 |
| BPPH | 379.1 → 208.8 | -30 | -48 | 379.1 → 192.9 | -30 | -86 |
| BPS | 249.8 → 108.0 | -17 | -36 | 249.8 → 109.0 | -16 | -42 |
| BPZ | 267.1 → 173.2 | -32 | -37 | 267.1 → 145.0 | -32 | -47 |
| ATBC | 403.2 → 128.9 | 26 | 25 | 403.2 → 185.2 | 26 | 35 |
| ATEC | 319.3 → 273.1 | 28 | 25 | 319.3 → 157.2 | 28 | 9 |
| BTHC | 515.4 → 413.2 | 37 | 26 | 515.4 → 213.0 | 19 | 13 |
| TBC | 361.5 → 185.1 | 26 | 25 | 361.5 → 129.1 | 26 | 35 |
| TEC | 277.3 → 157.1 | 62 | 19 | 277.3 → 203.1 | 57 | 11 |
| TPIB | 217.4 → 69.1 | 54 | 20 | 217.4 → 111.1 | 43 | 11 |
| TXIB | 287.4 → 199.2 | 56 | 7 | 287.4 → 111.2 | 56 | 16 |
| BHA | 179.0 → 149.0 | -40 | -28 | 179.0 → 107.7 | -40 | -42 |
| BHT | 219.3 → 203.0 | -80 | -38 | 219.3 → 163.1 | -80 | -34 |
| BHT-CHO | 233.2 → 217.2 | -90 | -45 | 233.1 → 175.1 | -81 | -45 |
| BHT-COOH | 249.0 → 205.2 | -25 | -30 | 249.0 → 189.0 | -25 | -40 |
| BHT-OH | 234.9 → 217.2 | -55 | -40 | 234.9 → 160.1 | -55 | -50 |
| BHT-Q | 220.1 → 205.1 | -100 | -33 | 220.1 → 148.0 | -100 | -41 |
| BHT-quinol | 235.1 → 220.1 | -90 | -24 | 235.1 → 205.1 | -90 | -20 |
| BMDM | 311.2 → 161.1 | 46 | 31 | 311.2 → 135.2 | 46 | 31 |
| EHS | 249.2 → 137.0 | -91 | -24 | 249.2 → 93.0 | -91 | -66 |
| HMS | 261.2 → 93.1 | -100 | -38 | 261.2 → 137.0 | -101 | -25 |
| IAMC | 249.2 → 161.0 | 90 | 20 | 249.2 → 76.7 | 70 | 70 |
| 4-MBC | 255.2 → 105.1 | 70 | 70 | 255.2 → 43.0 | 70 | 40 |
| ODPABA | 278.2 → 151.0 | 70 | 40 | 278.2 → 166.0 | 70 | 30 |
| OC | 362.2 → 232.3 | 12 | 26 | 362.2 → 279.3 | 12 | 34 |
| OMC | 291.2 → 160.9 | 12 | 23 | 291.2 → 179.5 | 12 | 14 |
| Cyanox 1212 | 600.3 → 414.3 | 14 | 20 | 600.3 → 330.3 | 14 | 22 |
| Cyanox 1790 | 717.2 → 191.2 | 51 | 37 | 717.2 → 135.2 | 51 | 81 |
| Ethanox 703 | 264.2 → 219.2 | 14 | 34 | 264.2 → 203.1 | 35 | 38 |
| Irganox 1035 | 660.5 → 249.2 | 108 | 45 | 660.5 → 193.0 | 108 | 61 |
| Irganox 1081 | 357.1 → 163.2 | -70 | -50 | 357.1 → 193.9 | -70 | -50 |
| Irganox 1098 | 637.5 → 525.4 | 22 | 35 | 637.5 → 581.4 | 25 | 31 |
| Irganox 245 | 587.5 → 177.2 | 80 | 71 | 587.5 → 263.0 | 80 | 29 |
| Irganox 259 | 656.5 → 415.1 | 20 | 25 | 656.5 → 471.3 | 20 | 34 |
| Irganox 330 | 774.3 → 219.2 | 81 | 41 | 774.3 → 511.9 | 81 | 69 |
| Irganox 445 | 406.2 → 91.1 | 45 | 35 | 406.2 → 192.8 | 45 | 35 |
| Irganox 565 | 589.4 → 250.2 | 60 | 60 | 589.4 → 289.2 | 70 | 60 |
| Irganox 697 | 714.5 → 307.0 | 15 | 50 | 714.5 → 159.0 | 30 | 69 |
| AO1024 | 551.3 → 73.0 | -40 | -45 | 551.3 → 115.0 | -30 | -60 |
| AO1222 | 355.2 → 137.0 | -35 | -30 | 355.2 → 108.1 | -39 | -30 |
| AO168 | 647.6 → 235.1 | 12 | 47 | 647.6 → 147.3 | 12 | 71 |
| AO2246 | 339.1 → 163.2 | -35 | -40 | 339.1 → 147.2 | -35 | -50 |
| AO22E46 | 456.5 → 233.2 | 12 | 19 | 456.5 →177.1 | 64 | 39 |
| AO4426 | 423.3 → 407.4 | -140 | 35 | 423.3 → 393.0 | -59 | 40 |
| AO44B25 | 381.4 → 163.1 | -11 | -39 | 381.4 → 338.3 | -26 | 35 |
| BBOT | 431.2 → 399.0 | 60 | 80 | 431.2 → 385.2 | 60 | 80 |
| DBHA | 214.2 → 90.9 | 5 | 15 | 214.2 → 106.1 | 38 | 16 |
| 2,4DtBP | 205.1 → 189.1 | -100 | 34 | 205.1 → 173.1 | -100 | 18 |
| DET | 133.2 → 88.1 | 38 | 13 | 133.2 → 46.2 | 20 | 18 |
| DPPD | 261.2 → 184.2 | 43 | 35 | 261.2 → 169.2 | 35 | 30 |
| 3,5-DTBH | 478.4 → 461.4 | 22 | 12 | 478.4 → 405.4 | 15 | 16 |
| DTBNP | 350.4 → 127.1 | 23 | 14 | 350.4 → 204.7 | 23 | 7 |
| DTG | 240.1 → 108.1 | 34 | 28 | 240.1 → 133.2 | 25 | 28 |
| DPG | 212.2 → 94.2 | 17 | 27 | 212.2 → 119.1 | 17 | 26 |
| MBEBP | 367.2 → 177.2 | -35 | -30 | 367.2 → 162.2 | -35 | -40 |
| MMBI | 162.9 → 131.0 | -35 | -30 | 162.9 → 114.8 | -35 | -40 |
| NPN | 220.1 → 142.0 | 51 | 55 | 220.1 → 115.1 | 51 | 31 |
| 4-tOP | 205.2 → 133.1 | -90 | -30 | 205.2 → 93.0 | -90 | -62 |
| TBBC | 357.1 → 193.9 | -70 | -90 | 357.1 → 179.1 | -70 | -60 |
| **Surrogate and internal standards** | | | | | | |
| DBP-d4 | 283.1 → 153.1 | 36 | 19 |  |  |  |
| DBzP-d4 | 351.0 → 91.0 | 41 | 33 |  |  |  |
| DEP-d4 | 277.1 → 153.1 | 16 | 21 |  |  |  |
| DEHP-d4 | 395.2 → 153.2 |  | 25 |  |  |  |
| DHxP-d4 | 339.0 → 153.2 | 26 | 21 |  |  |  |
| DMP-d4 | 199.1 → 167.1 | 11 | 15 |  |  |  |
| DOP-d4 | 395.2 → 153.2 | 40 | 50 | 395.2 → 69.1 | 40 | 50 |
| DPeP-d4 | 311.2 → 153.0 | 26 | 19 |  |  |  |
| DPrP-d4 | 255.1 → 153.1 | 21 | 17 |  |  |  |
| MBP-d4 | 224.8 → 81.1 | -30 | -20 |  |  |  |
| MBzP-d4 | 259.0 → 77.0 | -30 | -21 |  |  |  |
| MEP-d4 | 197.0 → 81.0 | -36 | -21 | 197.0 → 125.0 | -25 | -15 |
| MEHP-d4 | 379.3 → 137.3 | 30 | 40 |  |  |  |
| DEHA-d8 | 379.3 → 137.3 | 30 | 40 |  |  |  |
| DEHT-d4 | 395.2 → 153.2 |  | 25 |  |  |  |
| d-TBP | 294.2 → 101.9 | 80 | 26 | 294.2 → 83.0 | 80 | 77 |
| M6TBOEP | 405.1 → 201.0 | 90 | 21 |  |  |  |
| d-TCEP | 297.0 → 66.9 | 90 | 35 |  |  |  |
| d-TDCPP | 446.0 → 101.9 | 90 | 34 |  |  |  |
| d-TEP | 198.1 → 102.1 | 60 | 30 | 198.1 → 134.0 | 60 | 20 |
| d-TPP | 342.0 → 82.0 | 180 | 60 |  |  |  |
| tert-paraben-d9 | 202.0 → 136.0 | -30 | -19 | 202.0 → 192.0 | -30 | -19 |
| 2,4-OH-BP-13C6 | 218.0 → 91.0 | -40 | -30 |  |  |  |
| BTH-d4 | 140.0 → 113.1 | 35 | 30 |  |  |  |
| 1-H-BTR-d4 | 124.1 → 69.1 | 40 | 50 |  |  |  |
| 5-Me-BTR-d6 | 140.2 → 81.1 | 40 | 50 |  |  |  |
| BPA-d6 | 232.9 → 138.1 | -74 | -37 |  |  |  |
| BPS-C12 | 261.0 → 114.0 | -36 | -35 | 261.0 → 98.0 | -36 | -50 |
| BHT-d21 | 223..9 → 219.0 | -90 | -38 |  |  |  |
| DCDE | 513.9 → 443.8 |  | 23 | 513.9 → 441.8 |  | 23 |
| BPA-d16 | 241.0 → 142.2 | -120 | -40 | 241.0 → 142.2 | -120 | -40 |
| Coumaphos-d10 | 373.0 → 228.0 | 100 | 34 |  |  |  |

**Table S5.** Data of quality assurance and control tests, including the limit of quantification (LOQ, ng or ng/m^3^), recoveries (mean ± standard deviation), matrix effects (%) and blank contamination, and the detection frequency (DF) and measured concentrations of individual analytes in PM_2.5_ samples.

| **Plastic additives** | **LOQ (ng)^a^** | **LOQ (ng/m^3^)** | **Recovery^a^** | **Matrix effect** | **DF (%)** | **Concentration (ng/m^3^)** | | |
| --- | --- | --- | --- | --- | --- | --- | --- | --- |
|  |  |  |  |  |  | **min** | **median** | **max** |
| BBzP | 0.13 | 0.004 | 90.4%±6.6% | 140.8%±24.0% | 100 | 0.01 | 0.024 | 0.071 |
| iBCHP | 0.12 | 0.003 | 83.6%±5.8% | 134.5%±31.6% | 10 | <LOQ | <LOQ | 0.008 |
| BMPP | 0.17 | 0.005 | 61.2%±13.2% | 113.1%±7.5% | 100 | 0.018 | 0.057 | 0.45 |
| DAlP | 0.86 | 0.026 | 62.4%±16.3% | 96.1%±37.7% | 5 | <LOQ | <LOQ | 0.79 |
| DAmP | 0.11 | 0.003 | 87.1%±7.1% | 111.4%±31.3% | 83 | <LOQ | 0.007 | 0.017 |
| DiBP | 49.1 | 1.50 | 81.8%±21.0% | 102.6%±27.5% | 97 | <LOQ | 11.1 | 32.1 |
| DBP | 111 | 3.40 | 78.4%±22.6% | 126.0%±11.0% | 100 | 5.30 | 15.3 | 33.3 |
| DBzP | 0.009 | 0.0003 | 77.0%±4.5% | 148.2%±30.0% | 30 | <LOQ | <LOQ | 0.007 |
| DEP | 9.44 | 0.289 | 79.9%±11.3% | 102.5%±37.0% | 0 | <LOQ | <LOQ | <LOQ |
| DEHP | 27.3 | 0.837 | 118.2%±22.0% | 65.4%±19.6% | 100 | 5.58 | 28.8 | 241 |
| DiHeP | 0.11 | 0.003 | 70.5%±17.6% | 109.7%±5.8% | 100 | 0.010 | 0.029 | 0.100 |
| DiHxP | 0.18 | 0.006 | 72.2%±12.1% | 109.2%±17.8% | 100 | 0.024 | 0.071 | 0.720 |
| DHxP | 0.04 | 0.001 | 47.3%±21.3% | 94.6%±15.3% | 0 | <LOQ | <LOQ | <LOQ |
| DMiP | 2.91 | 0.089 | 82.1%±18.5% | 102.2%±44.4% | 80 | <LOQ | 0.205 | 0.620 |
| DMP | 6.89 | 0.207 | 70.4%±10.1% | 101.6%±12.4% | 0 | <LOQ | <LOQ | <LOQ |
| DNP/DiNP**^d^** | 19.2 | 0.578 | 65.7%±5.8% | 146.7%±20.7% | 100 | 0.92 | 2.37 | 30.2 |
| DiPeP | 0.13 | 0.004 | 83.9%±5.2% | 111.4%±31.3% | 20 | <LOQ | <LOQ | 0.007 |
| DPHiP | 0.16 | 0.005 | 82.1%±4.1% | 108.8%±12.5% | 0 | <LOQ | <LOQ | <LOQ |
| DPHP | 0.51 | 0.016 | 83.9%±5.2% | 130.5%±29.3% | 0 | <LOQ | <LOQ | <LOQ |
| DiPrP | 3.52 | 0.107 | 79.0%±10.5% | 121.1%±10.6% | 0 | <LOQ | <LOQ | <LOQ |
| DPrP | 3.48 | 0.107 | 86.4%±8.2% | 104.9%±5.1% | 0 | <LOQ | <LOQ | <LOQ |
| DUP | 0.06 | 0.002 | 82.2%±14.3% | 82.2%±14.3% | 80 | <LOQ | 0.009 | 0.085 |
| MBP/MiBP**^d^** | 2.17 | 0.067 | 75.9%±6.4% | 115.2%±4.3% | 100 | 0.19 | 0.622 | 2.18 |
| MBzP | 0.05 | 0.001 | 77.4%±8.2% | 124.9%±5.1% | 89 | <LOQ | 0.006 | 0.018 |
| MCHP | 0.11 | 0.003 | 95.4%±7.7% | 120.0%±8.5% | 9 | <LOQ | <LOQ | 0.061 |
| MEP | 0.27 | 0.008 | 108.8%±15.5% | 126.5%±9.9% | 100 | 0.025 | 0.092 | 0.190 |
| MEHP | 1.25 | 0.038 | 88.6%±7.7% | 188.6%±22.2% | 100 | 0.200 | 0.990 | 4.76 |
| MECPP | 0.044 | 0.001 | 81.2%±9.1% | 129.6%±12.0% | 89 | <LOQ | 0.010 | 0.040 |
| MEHHP | 0.093 | 0.003 | 93.0%±12.5% | 98.8%±11.8% | 100 | 0.017 | 0.130 | 0.480 |
| MEOHP | 0.033 | 0.001 | 93.0%±12.5% | 120.5%±2.1% | 100 | 0.011 | 0.087 | 0.490 |
| MHeP | 0.54 | 0.017 | 83.7%±11.3% | 122.8%±8.6% | 0 | <LOQ | <LOQ | <LOQ |
| MHxP | 0.07 | 0.002 | 84.1%±4.4% | 113.6%±7.1% | 100 | 0.002 | 0.004 | 0.009 |
| MMP | 0.99 | 0.030 | 119.3%±2.6% | 129.3%±2.6% | 100 | 0.230 | 2.29 | 9.13 |
| MiNP | 0.24 | 0.007 | 108.7%±7.6% | 135.3%±7.8% | 100 | 0.053 | 0.130 | 0.640 |
| MOP | 1.19 | 0.036 | 117.6%±10.1% | 150.7%±16.1% | 100 | 0.220 | 0.930 | 5.61 |
| MPeP | 0.052 | 0.002 | 83.1%±7.0% | 117.6%±10.1% | 40 | <LOQ | <LOQ | 0.009 |
| MiPrP | 0.058 | 0.002 | 79.4%±10.2% | 113.2%±6.7% | 80 | <LOQ | 0.013 | 0.037 |
| BARO | 0.24 | 0.007 | 68.0%±32.2% | 107.8%±25.9% | 0 | <LOQ | <LOQ | <LOQ |
| BRO | 0.2 | 0.006 | 63.6%±24.0% | 92.6%±5.2% | 0 | <LOQ | <LOQ | <LOQ |
| BO | 19.5 | 0.599 | 85.0%±11.0% | 90.7%±19.0% | 0 | <LOQ | <LOQ | <LOQ |
| DBF | 1.04 | 0.032 | 78.5%±6.0% | 96.7%±5.8% | 40 | <LOQ | <LOQ | 0.190 |
| DBS | 0.37 | 0.011 | 65.1%±12.3% | 67.4%±13.6% | 100 | 0.018 | 0.040 | 0.140 |
| DEHM | 0.38 | 0.012 | 79.1%±21.3% | 56.0%±10.3% | 100 | 0.049 | 0.083 | 1.62 |
| DES | 0.55 | 0.017 | 70.2%±7.7% | 182.0%±17.0% | 0 | <LOQ | <LOQ | <LOQ |
| DMS | 0.33 | 0.010 | 66.4%±10.0% | 120.5%±8.4% | 70 | <LOQ | 0.023 | 0.22 |
| EHSe | 0.31 | 0.010 | 115.8%±8.5% | 113.4%±3.5% | 100 | 0.062 | 0.242 | 8.46 |
| GMO | 11.0 | 0.321 | 53.3%±17.3% | 79.3%±8.5% | 80 | <LOQ | 1.30 | 12.3 |
| MO | 17.5 | 0.541 | 80.1%±35.6% | 152.6%±28.1% | 55 | <LOQ | 0.615 | 4.29 |
| MOA | 0.71 | 0.022 | 44.3%±17.9% | 80.1%±35.6% | 0 | <LOQ | <LOQ | <LOQ |
| IPM | 3.65 | 0.109 | 30.0%±1.8% | 114.1%±21.7% | 80 | <LOQ | 0.261 | 0.950 |
| IPP | 1.24 | 0.038 | 98.8%±25.9% | 147.0%±23.6% | 100 | 0.040 | 0.248 | 0.750 |
| PO | 6.74 | 0.207 | 65.3%±19.1% | 99.9%±10.4% | 0 | <LOQ | <LOQ | <LOQ |
| THPO | 0.051 | 0.002 | 79.7%±14.7% | 157.2%±32.6% | 0 | <LOQ | <LOQ | <LOQ |
| DEHT | 25.1 | 0.770 | 105.3%±48.6% | 65.4%±19.6% | 97 | <LOQ | 16.5 | 115 |
| DEGDB | 0.27 | 0.008 | 88.9%±11.7% | 101.4%±3.7% | 100 | 0.013 | 0.039 | 1.54 |
| DPGDB | 0.56 | 0.017 | 114.4%±4.9% | 107.6%±8.6% | 97 | <LOQ | 0.050 | 1.74 |
| DINCH | 8.56 | 0.263 | 57.3%±9.1% | 104.9%±4.5% | 55 | <LOQ | 0.331 | 4.22 |
| DiDEAZ | 2.44 | 0.075 | 91.4%±12.3% | 91.4%±5.0% | 0 | <LOQ | <LOQ | <LOQ |
| DHAZ | 0.18 | 0.005 | 118.3%±6.4% | 82.4%±16.8% | 0 | <LOQ | <LOQ | <LOQ |
| DMAZ | 1.95 | 0.060 | 78.7%±14.4% | 128.1%±8.0% | 64 | <LOQ | 0.167 | 0.360 |
| DiOAZ | 0.15 | 0.004 | 156.0%±10.6% | 114.4%±4.9% | 75 | <LOQ | 0.013 | 0.050 |
| TCaT | 2.34 | 0.072 | 33.9%±9.7% | 156.9%±15.3% | 94 | <LOQ | 0.360 | 7.65 |
| THTM | 0.02 | 0.001 | 65.2%±30.1% | 147.0%±50.0% | 6 | <LOQ | <LOQ | 0.008 |
| TiNTM | 0.38 | 0.012 | 75.4%±6.8% | 32.4%±0.3% | 45 | <LOQ | <LOQ | 1.06 |
| TOTM | 1.32 | 0.040 | 34.6%±7.2% | 138.1%±7.7% | 100 | 0.050 | 0.390 | 7.68 |
| TiTDM | 45.1 | 1.38 | 75.6%±5.6% | 121.4%±12.3% | 60 | <LOQ | <LOQ | 6.85 |
| TBP | 8.49 | 0.261 | 81.3%±10.5% | 119.2%±8.2% | 100 | 0.285 | 1.12 | 6.26 |
| TBOEP | 0.19 | 0.006 | 80.2%±7.8% | 92.7%±8.4% | 89 | <LOQ | 0.044 | 1.64 |
| TCEP | 6.48 | 0.199 | 81.8%±6.8% | 125.6%±15.7% | 100 | 0.370 | 0.740 | 3.11 |
| TCIPP | 46.8 | 1.44 | 78.1%±10.1% | 112.8%±12.7% | 100 | 2.3 | 4.37 | 8.42 |
| TDBPP | 0.18 | 0.006 | 79.8%±5.1% | 85.9%±7.9% | 25 | <LOQ | <LOQ | 0.13 |
| TDCPP | 5.58 | 0.172 | 78.7%±7.0% | 79.2%±4.8% | 100 | 0.370 | 0.5 | 1.49 |
| TEP | 0.72 | 0.022 | 85.3%±8.5% | 138.6%±15.2% | 89 | <LOQ | 0.086 | 2.98 |
| TEHP | 0.13 | 0.004 | 71.5%±17.7% | 125.4%±34.2% | 100 | 0.022 | 0.036 | 0.99 |
| TPrP | 0.54 | 0.017 | 78.6%±8.3% | 129.6%±14.3% | 0 | <LOQ | <LOQ | <LOQ |
| V6 | 0.65 | 0.020 | 88.5%±8.8% | 122.3%±13.6% | 0 | <LOQ | <LOQ | <LOQ |
| BPADP | 0.08 | 0.003 | 76.9%±5.5% | 70.7%±9.7% | 94 | <LOQ | 0.006 | 0.060 |
| CDP | 0.25 | 0.008 | 87.6%±7.8% | 95.1%±7.6% | 80 | <LOQ | 0.021 | 0.350 |
| DDPP | 0.059 | 0.002 | 93.3%±5.4% | 114.2%±12.8% | 86 | <LOQ | 0.004 | 0.230 |
| EHDPP | 1.04 | 0.032 | 90.4%±18.0% | 126.1%±16.3% | 50 | <LOQ | <LOQ | 3.15 |
| RDP | 0.06 | 0.002 | 79.5%±7.6% | 68.5%±7.1% | 55 | <LOQ | 0.008 | 0.06 |
| TCrP | 0.98 | 0.031 | 82.8%±7.4% | 125.2%±19.6% | 100 | 0.034 | 0.23 | 2.14 |
| T34DMPP | 0.035 | 0.001 | 84.7%±5.8% | 145.7%±39.7% | 94 | <LOQ | 0.005 | 0.067 |
| T35DMPP | 0.031 | 0.001 | 84.6%±5.0% | 138.0%±19.1% | 100 | 0.001 | 0.005 | 0.046 |
| TPHP | 0.36 | 0.011 | 85.4%±8.1% | 93.6%±6.7% | 100 | 0.049 | 0.120 | 2.46 |
| B2tBPPP | 0.016 | 0.001 | 87.1%±10.3% | 114.3%±27.2% | 0 | <LOQ | <LOQ | <LOQ |
| B4tBPPP | 1.14 | 0.035 | 85.4%±3.6% | 125.4%±7.6% | 0 | <LOQ | <LOQ | <LOQ |
| 2tBPDPP | 0.35 | 0.011 | 75.2%±6.0% | 76.8%±8.8% | 0 | <LOQ | <LOQ | <LOQ |
| 4tBPDPP | 0.085 | 0.003 | 73.1%±8.5% | 130.4%±17.3% | 100 | 0.007 | 0.022 | 0.26 |
| 24DIPPDPP | 0.088 | 0.003 | 87.8%±12.6% | 136.2%±19.0% | 100 | 0.003 | 0.013 | 0.100 |
| B24DIPPPP | 0.012 | 0.0004 | 77.4%±4.4% | 77.8%±8.9% | 97 | <LOQ | 0.001 | 0.011 |
| B2IPPPP | 0.044 | 0.001 | 85.3%±10.2% | 132.5%±4.9% | 100 | 0.003 | 0.010 | 0.051 |
| B4IPPPP | 0.024 | 0.001 | 86.5%±8.6% | 119.1%±21.5% | 100 | 0.001 | 0.003 | 0.017 |
| 2IPPDPP | 0.13 | 0.004 | 76.1%±4.9% | 85.1%±9.4% | 100 | 0.006 | 0.043 | 0.310 |
| 4IPPDPP | 0.041 | 0.001 | 83.8%±9.8% | 83.0%±9.3% | 100 | 0.002 | 0.013 | 0.100 |
| T4tBPP | 0.018 | 0.001 | 74.8%±3.3% | 77.2%±9.1% | 86 | <LOQ | 0.002 | 0.006 |
| T2IPPP | 0.056 | 0.002 | 90.7%±7.3% | 146.8%±5.7% | 97 | <LOQ | 0.006 | 0.048 |
| T3IPPP | 0.029 | 0.001 | 71.3%±15.8% | 128.8%±29.6% | 94 | <LOQ | 0.003 | 0.023 |
| T4IPPP | 0.017 | 0.001 | 71.6%±16.8% | 133.9%±27.4% | 0 | <LOQ | <LOQ | <LOQ |
| DnBA/DiBA**^d^** | 6.81 | 0.209 | 79.0%±8.8% | 152.2%±22.9% | 94 | <LOQ | 3.11 | 13.1 |
| DCaA | 2.25 | 0.069 | 106.9%±15.3% | 103.0%±7.9% | 100 | 0.240 | 0.870 | 10.2 |
| DiDeA | 0.41 | 0.012 | 88.2%±19.2% | 80.6%±3.3% | 91 | <LOQ | 0.041 | 0.740 |
| DEA | 0.52 | 0.016 | 85.7%±12.5% | 114.3%±15.4% | 0 | <LOQ | <LOQ | <LOQ |
| DEHA | 25.4 | 0.779 | 73.0%±11.5% | 95.8%±13.2% | 97 | <LOQ | 2.80 | 20.2 |
| DHeNoA | 18.0 | 0.552 | 87.6%±14.8% | 101.8%±16.8% | 97 | <LOQ | 1.82 | 14.5 |
| DMA | 4.56 | 0.140 | 46.7%±7.1% | 100.5%±9.0% | 25 | <LOQ | <LOQ | 0.4 |
| DiNA | 0.49 | 0.015 | 102.2%±22.9% | 96.7%±5.3% | 100 | 0.016 | 0.037 | 0.670 |
| Benzil | 1.04 | 0.032 | 84.0%±11.7% | 109.1%±45.3% | 45 | <LOQ | <LOQ | 0.190 |
| BP | 28.5 | 0.874 | 79.8%±14.4% | 102.7%±54.0% | 0 | <LOQ | <LOQ | <LOQ |
| DETX | 0.17 | 0.005 | 66.7%±9.8% | 127.3%±4.9% | 80 | <LOQ | 0.044 | 0.160 |
| DMAB | 0.18 | 0.006 | 66.8%±2.1% | 97.7%±35.2% | 64 | <LOQ | 0.012 | 0.100 |
| EAB | 0.32 | 0.010 | 84.0%±13.7% | 87.9%±17.7% | 79 | <LOQ | 0.053 | 0.230 |
| EAQ | 4.59 | 0.141 | 83.7%±11.6% | 61.0%±7.4% | 0 | <LOQ | <LOQ | <LOQ |
| EDMAB | 0.15 | 0.005 | 78.4%±8.1% | 92.9%±43.8% | 75 | <LOQ | 0.040 | 0.250 |
| 2-ITX | 0.19 | 0.006 | 89.7%±8.7% | 126.7%±4.9% | 40 | <LOQ | <LOQ | 0.620 |
| MBB | 0.73 | 0.022 | 76.1%±8.0% | 186.8%±23.1% | 94 | <LOQ | 0.120 | 0.510 |
| 4-MBP | 0.99 | 0.030 | 72.6%±8.4% | 81.8%±40.4% | 0 | <LOQ | <LOQ | <LOQ |
| MEK | 0.03 | 0.001 | 70.8%±4.5% | 69.6%±20.8% | 25 | <LOQ | <LOQ | 0.005 |
| MK | 0.11 | 0.004 | 63.7%±1.4% | 98.5%±19.3% | 97 | <LOQ | 0.039 | 0.450 |
| PBZ | 0.08 | 0.002 | 83.9%±7.5% | 32.9%±23.4% | 100 | 0.011 | 0.053 | 0.230 |
| PI-184 | 2.61 | 0.080 | 94.3%±16.8% | 124.9%±34.9% | 60 | <LOQ | 0.236 | 2.50 |
| PI-651 | 0.56 | 0.017 | 76.3%±9.4% | 92.1%±33.1% | 10 | <LOQ | <LOQ | 0.110 |
| BP1 | 0.017 | 0.001 | 88.8%±9.3% | 187.6%±10.4% | 94 | 0.001 | 0.008 | 0.026 |
| BP2 | 0.075 | 0.002 | 75.1%±14.5% | 167.2%±11.4% | 0 | <LOQ | <LOQ | <LOQ |
| BP3 | 0.18 | 0.006 | 95.6%±8.3% | 45.8%±3.7% | 89 | <LOQ | 0.023 | 0.074 |
| BP4 | 0.99 | 0.030 | 89.9%±11.7% | 119.9%±11.7% | 0 | <LOQ | <LOQ | <LOQ |
| BP6 | 7.35 | 0.226 | 83.6%±5.6% | 142.4%±11.6% | 0 | <LOQ | <LOQ | <LOQ |
| BP8 | 0.044 | 0.001 | 78.0%±8.2% | 167.4%±15.5% | 5 | <LOQ | <LOQ | 0.012 |
| 4-OH-BP | 0.21 | 0.007 | 81.9%±10.3% | 179.5%±12.8% | 54 | <LOQ | 0.012 | 0.044 |
| 2,3,4-OH-BP | 73.5 | 2.26 | 99.8%±81.8% | 122.9%±2.3% | 0 | <LOQ | <LOQ | <LOQ |
| BTH | 12.2 | 0.368 | 92.4%±15.5% | 178.9%±9.7% | 5 | <LOQ | <LOQ | 0.620 |
| 2-Cl-BTH | 179 | 5.478 | 101.0%±12.5% | 98.8%±4.3% | 0 | <LOQ | <LOQ | <LOQ |
| 2-Me-BTH | 0.19 | 0.006 | 67.0%±10.2% | 118.3%±5.0% | 97 | <LOQ | 0.026 | 0.130 |
| 2-Me-S-BTH | 14.5 | 0.445 | 81.7%±10.8% | 65.8%±39.5% | 30 | <LOQ | <LOQ | 1.10 |
| 2-Mo-BTH | 0.58 | 0.018 | 75.5%±3.7% | 145.2%±14.3% | 0 | <LOQ | <LOQ | <LOQ |
| 2NH_2_-BTH | 0.51 | 0.016 | 56.0%±6.9% | 97.0%±9.5% | 45 | <LOQ | <LOQ | 0.150 |
| 2-OH-BTH | 6.29 | 0.193 | 51.5%±8.1% | 129.8%±12.3% | 0 | <LOQ | <LOQ | <LOQ |
| UV-P | 1.21 | 0.037 | 70.9%±5.4% | 89.5%±30.3% | 0 | <LOQ | <LOQ | <LOQ |
| UV-PS | 0.18 | 0.006 | 57.8%±15.2% | 97.4%±11.6% | 0 | <LOQ | <LOQ | <LOQ |
| UV-234 | 0.042 | 0.001 | 68.4%±5.6% | 100.1%±13.0% | 75 | <LOQ | 0.007 | 0.160 |
| UV-320 | 0.74 | 0.023 | 65.4%±7.7% | 107.0%±6.9% | 0 | <LOQ | <LOQ | <LOQ |
| UV-326 | 0.42 | 0.013 | 69.0%±13.0% | 94.6%±7.0% | 50 | <LOQ | <LOQ | 0.670 |
| UV-327 | 0.004 | 0.0001 | 59.6%±4.4% | 93.0%±4.0% | 94 | <LOQ | 0.003 | 0.030 |
| UV-328 | 0.009 | 0.0003 | 71.3%±7.3% | 90.0%±3.9% | 97 | <LOQ | 0.006 | 0.038 |
| UV-350 | 0.35 | 0.011 | 74.7%±6.5% | 95.7%±6.3% | 0 | <LOQ | <LOQ | <LOQ |
| UV-360 | 0.11 | 0.004 | 69.4%±69.3% | 122.9%±2.3% | 40 | <LOQ | <LOQ | 0.016 |
| 5-Cl-BTR | 8.54 | 0.262 | 88.2%±12.9% | 152.5%±19.8% | 94 | <LOQ | 1.7 | 40.2 |
| 1-H-BTR | 33.3 | 1.02 | 67.2%±18.6% | 131.5%±2.6% | 0 | <LOQ | <LOQ | <LOQ |
| 1-OH-BTR | 76.9 | 2.36 | 62.2%±35.9% | 147.5%±10.6% | 0 | <LOQ | <LOQ | <LOQ |
| 4-Me-1-H-BTR | 1.21 | 0.037 | 84.2%±13.2% | 148.6%±9.5% | 50 | <LOQ | <LOQ | 0.79 |
| 5-Me-1-H-BTR | 1.65 | 0.051 | 84.2%±13.2% | 117.6%±94.5% | 89 | <LOQ | 0.26 | 0.97 |
| 5,6-2Me-1-BTR | 22.0 | 0.674 | 73.0%±5.3% | 129.3%±16.2% | 0 | <LOQ | <LOQ | <LOQ |
| BPA | 1.33 | 0.041 | 80.6%±6.0% | 131.3%±17.3% | 100 | 0.18 | 1.1 | 10.6 |
| BPAF | 0.01 | 0.0003 | 67.2%±43.2% | 99.7%±11.5% | 100 | 0.001 | 0.004 | 0.18 |
| BPAP | 5.11 | 0.153 | 68.3%±7.0% | 145.4%±15.7% | 0 | <LOQ | <LOQ | <LOQ |
| BPB | 4.35 | 0.133 | 55.3%±11.3% | 107.4%±9.1% | 0 | <LOQ | <LOQ | <LOQ |
| BPBP | 1.82 | 0.056 | 60.3%±7.7% | 131.6%±10.1% | 0 | <LOQ | <LOQ | <LOQ |
| BPC | 55.6 | 1.70 | 55.3%±11.3% | 109.5%±6.0% | 0 | <LOQ | <LOQ | <LOQ |
| BPE | 27.2 | 0.829 | 74.1%±6.5% | 165.3%±15.2% | 0 | <LOQ | <LOQ | <LOQ |
| BPF | 0.66 | 0.020 | 56.3%±5.2% | 150.4%±16.8% | 100 | 0.061 | 0.54 | 7.00 |
| BPG | 0.31 | 0.010 | 59.4%±6.2% | 69.4%±9.1% | 5 | <LOQ | <LOQ | 0.12 |
| BPM | 2.78 | 0.085 | 72.1%±6.3% | 91.0%±7.4% | 0 | <LOQ | <LOQ | <LOQ |
| BPP | 4.24 | 0.130 | 66.4%±6.3% | 82.5%±6.4% | 0 | <LOQ | <LOQ | <LOQ |
| BPPH | 24.4 | 0.748 | 66.9%±9.3% | 111.8%±9.9% | 0 | <LOQ | <LOQ | <LOQ |
| BPS | 0.51 | 0.016 | 83.7%±15.2% | 160.5%±11.4% | 94 | <LOQ | 0.024 | 0.19 |
| BPZ | 2.86 | 0.088 | 56.2%±7.4% | 147.5%±13.6% | 0 | <LOQ | <LOQ | <LOQ |
| ATBC | 14.3 | 0.437 | 93.4%±9.1% | 87.6%±14.8% | 100 | 0.59 | 5.25 | 22.9 |
| ATEC | 0.19 | 0.006 | 79.6%±6.3% | 107.2%±20.2% | 0 | <LOQ | <LOQ | <LOQ |
| BTHC | 0.17 | 0.005 | 112.8%±27.9% | 139.1%±12.4% | 100 | 0.006 | 0.014 | 0.078 |
| TBC | 4.05 | 0.124 | 64.6%±7.1% | 112.5%±6.1% | 100 | 0.13 | 0.47 | 6.34 |
| TEC | 1.13 | 0.035 | 68.3%±5.5% | 117.8%±3.0% | 100 | 0.12 | 0.72 | 5.35 |
| TPIB | 33.1 | 1.01 | 40.7%±9.2% | 86.8%±8.3% | 94 | <LOQ | 9.1 | 84.1 |
| TXIB | 46.5 | 1.43 | 50.6%±8.1% | 90.3%±11.6% | 83 | <LOQ | 3.4 | 63.9 |
| BHA | 3.72 | 0.114 | 104.0%±9.5% | 59.5%±15.9% | 0 | <LOQ | <LOQ | <LOQ |
| BHT | 1.61 | 0.049 | 109.3%±36.8% | 50.0%±25.8% | 97 | 0.16 | 0.56 | 1.90 |
| BHT-CHO | 4.52 | 0.139 | 99.7%±28.1% | 101.6%±4.2% | 100 | 0.56 | 3.30 | 8.40 |
| BHT-COOH | 0.21 | 0.007 | 96.9%±10.2% | 133.0%±5.3% | 97 | <LOQ | 0.073 | 0.13 |
| BHT-OH | 0.11 | 0.004 | 85.3%±13.8% | 80.0%±7.7% | 100 | 0.026 | 0.045 | 0.11 |
| BHT-Q | 34.2 | 1.05 | 54.4%±3.1% | 94.7%±10.5% | 20 | <LOQ | <LOQ | 8.63 |
| BHT-quinol | 33.4 | 1.03 | 50.4%±6.8% | 80.0%±5.0% | 10 | <LOQ | <LOQ | 2.30 |
| BMDM | 0.74 | 0.023 | 61.2%±36.5% | 36.6%±6.3% | 0 | <LOQ | <LOQ | <LOQ |
| EHS | 3.42 | 0.105 | 66.8%±15.4% | 96.3%±3.5% | 0 | <LOQ | <LOQ | <LOQ |
| HMS | 4.23 | 0.130 | 56.4%±10.0% | 80.6%±3.4% | 0 | <LOQ | <LOQ | <LOQ |
| IAMC | 1.43 | 0.044 | 76.1%±7.6% | 108.9%±7.9% | 0 | <LOQ | <LOQ | <LOQ |
| 4-MBC | 0.45 | 0.014 | 72.8%±9.4% | 102.4%±3.2% | 60 | <LOQ | 0.019 | 0.09 |
| ODPABA | 0.04 | 0.001 | 58.7%±10.9% | 103.1%±8.3% | 30 | <LOQ | <LOQ | 0.004 |
| OC | 0.36 | 0.011 | 66.1%±12.6% | 114.6%±8.3% | 100 | 0.020 | 0.063 | 0.230 |
| OMC | 0.45 | 0.014 | 54.1%±30.8% | 108.1%±16.1% | 100 | 0.022 | 0.075 | 0.220 |
| Cyanox 1212 | 17.4 | 0.534 | 71.4%±3.7% | 166.5%±4.2% | 0 | <LOQ | <LOQ | <LOQ |
| Cyanox 1790 | 1.81 | 0.055 | 65.9%±6.1% | 98.9%±6.1% | 0 | <LOQ | <LOQ | <LOQ |
| Ethanox 703 | 0.13 | 0.004 | 166.5%±4.2% | 75.2%±12.1% | 100 | 0.015 | 0.050 | 0.890 |
| Irganox 1035 | 0.081 | 0.002 | 60.5%±11.7% | 109.7%±28.1% | 0 | <LOQ | <LOQ | <LOQ |
| Irganox 1081 | 0.034 | 0.001 | 62.2%±6.4% | 62.2%±6.4% | 0 | <LOQ | <LOQ | <LOQ |
| Irganox 1098 | 0.21 | 0.007 | 59.2%±7.1% | 58.2%±22.7% | 5 | <LOQ | <LOQ | 0.0073 |
| Irganox 245 | 4.12 | 0.126 | 47.3%±9.5% | 107.7%±5.4% | 0 | <LOQ | <LOQ | <LOQ |
| Irganox 259 | 0.091 | 0.003 | 30.1%±4.4% | 141.8%±40.8% | 0 | <LOQ | <LOQ | <LOQ |
| Irganox 330 | 25.6 | 0.787 | 61.6%±3.7% | 118.8%±15.1% | 0 | <LOQ | <LOQ | <LOQ |
| Irganox 445 | 8.62 | 0.264 | 68.9%±6.1% | 53.6%±7.8% | 0 | <LOQ | <LOQ | <LOQ |
| Irganox 565 | 0.13 | 0.004 | 71.3%±8.7% | 104.9%±9.9% | 0 | <LOQ | <LOQ | <LOQ |
| Irganox 697 | 0.07 | 0.002 | 67.1%±12.9% | 75.1%±7.0% | 0 | <LOQ | <LOQ | <LOQ |
| AO1024 | 0.02 | 0.001 | 64.3%±0.8% | 76.3%±8.2% | 0 | <LOQ | <LOQ | <LOQ |
| AO1222 | 0.03 | 0.001 | 59.2%±6.4% | 69.3%±7.7% | 0 | <LOQ | <LOQ | <LOQ |
| AO168 | 9.89 | 0.304 | 69.2%±2.8% | 132.4%±56.3% | 0 | <LOQ | <LOQ | <LOQ |
| AO2246 | 1.12 | 0.034 | 60.5%±17.2% | 65.8%±8.4% | 0 | <LOQ | <LOQ | <LOQ |
| AO22E46 | 0.02 | 0.0005 | 54.6%±9.3% | 54.6%±9.3% | 20 | <LOQ | <LOQ | 0.004 |
| AO4426 | 8.93 | 0.274 | 62.0%±3.4% | 71.3%±8.7% | 0 | <LOQ | <LOQ | <LOQ |
| AO44B25 | 0.02 | 0.0006 | 75.1%±5.2% | 75.1%±5.2% | 0 | <LOQ | <LOQ | <LOQ |
| BBOT | 0.03 | 0.001 | 75.2%±15.1% | 168.6%±18.5% | 0 | <LOQ | <LOQ | <LOQ |
| DBHA | 0.48 | 0.015 | 67.8%±25.9% | 89.2%±14.9% | 0 | <LOQ | <LOQ | <LOQ |
| 2,4DtBP | 4.24 | 0.130 | 49.7%±26.0% | 101.9%±12.9% | 6 | <LOQ | <LOQ | 0.170 |
| DET | 2.89 | 0.089 | 79.0%±7.2% | 119.0%±7.2% | 0 | <LOQ | <LOQ | <LOQ |
| DPPD | 0.03 | 0.001 | 71.9%±4.8% | 116.2%±5.7% | 10 | <LOQ | <LOQ | 0.005 |
| 3,5-DTBH | 0.16 | 0.005 | 60.7%±16.0% | 102.0%±3.4% | 0 | <LOQ | <LOQ | <LOQ |
| DTBNP | 51.5 | 1.58 | 71.2%±5.7% | 122.4%±70.1% | 60 | <LOQ | 1.82 | 9.50 |
| DTG | 0.16 | 0.005 | 64.9%±10.6% | 89.8%±7.4% | 6 | <LOQ | <LOQ | 0.100 |
| DPG | 0.46 | 0.014 | 73.9%±18.3% | 82.8%±5.6% | 0 | <LOQ | <LOQ | <LOQ |
| MBEBP | 0.07 | 0.002 | 64.5%±19.6% | 87.1%±12.9% | 0 | <LOQ | <LOQ | <LOQ |
| MMBI | 44.4 | 1.36 | 84.4%±3.8% | 84.4%±3.8% | 0 | <LOQ | <LOQ | <LOQ |
| NPN | 1.52 | 0.047 | 62.1%±5.7% | 104.0%±11.9% | 79 | <LOQ | 0.13 | 2.01 |
| 4-tOP | 0.35 | 0.011 | 66.3%±16.9% | 117.6%±11.0% | 0 | <LOQ | <LOQ | <LOQ |
| TBBC | 1.25 | 0.038 | 83.8%±24.2% | 114.3%±11.4% | 0 | <LOQ | <LOQ | <LOQ |

**^a^** The LOQ of an analyte is defined as its response ten times the standard deviation of the noise if the analyte is not present in procedural blanks. For the chemicals detectable in blanks, their LOQs were determined as the average contamination in blanks plus ten times the standard deviation of average contamination;

**^b^** Recovery data were retrieved from the spiking tests, where known amounts of target analytes were spiked with pre-cleaned sodium sulfate and processed in six replicates with the analytical procedures detailed in the main text.

**^c^** Matrix effect was conducted with six replicates of pooled PM2.5 samples. The pooled samples were extracted without addition of any target analytes or surrogate standards. The final extract was reconstituted with 200 μL of methanol and then aliquoted into two sub-samples with equal volume (100 μL each). Sub-sample A was spiked with 100 μL of a standard mixture of analytes as well as surrogate standards. Sub-sample B was spiked with 100 μL of methanol. An external standard solution (S) was prepared by mixing the 100 μL of analyte mixtures (including surrogate standards) with 100 μL methanol. By comparing the response differences of the analytes in the sub-samples A and B to the responses of the analytes in the external standard, a matrix effect (ME) value was determined as:

$$\text{ME}\left( \text{\%} \right)\text{=100×}\frac{\text{(Ai-Bi)}}{\text{Si}}$$

where Ai, Bi and Si are the chromatographic peak areas of the analyte (i) in sub-samples A and B and external standard solution (S), respectively.

**^d^** Co-eluted and their concentrations were reported as a combination of two isomers.

**Table S6.** Groups of plastic additives with detection frequencies higher than 80% in PM_2.5_ samples.

| **Group** | | **Plastic additives ^a^** |
| --- | --- | --- |
| **G1** | phthalate esters (PAEs) | BBzP, BMPP, DAmP, DiBP, DBP, DEHP, DiHeP, DiHxP, DMiP, DNP/DiNP, DUP |
| **G2** | phthalate mono-esters (mono-PAEs) | MBP/MiBP, MBzP, MEP, MEHP, MECPP, MEHHP, MEOHP, MHxP, MMP, MiNP, MOP, MiPrP |
| **G3** | fatty acid ester (FAE) plasticizers | DBS, EHSe, DEHM, IPM, IPP, GMO |
| **G4** | other plasticizers | DEHT, DEGDB, DPGDB, TCaT, TOTM |
| **G5** | alkyl organophosphate esters (alkyl-OPEs) | TBP, TBOEP, TCIPP, TCEP, TDCPP, TEP, TEHP |
| **G6** | aryl organophosphate esters (aryl-OPEs) | BPADP, CDP, DDPP, TCrP, T34DMPP, T35DMPP, TPHP |
| **G7** | isopropylated and *tert*-butylated triarylphosphate esters (ITPs & TBPPs) | 4tBPDPP, 24DIPPDPP, B24DIPPPP, B2IPPPP, B4IPPPP, 2IPPDPP, 4IPPDPP, T4tBPP, T2IPPP, T3IPPP |
| **G8** | adipate esters (AEs) | DnBA/DiBA, DCaA, DiDeA, DEHA, DHeNoA, DiNA |
| **G9** | benzophenones and benzoates (BZPs & BZAs) | EAB, MBB, MK, PBZ, BP1, BP3 |
| **G10** | benzothiazoles and benzotriazoles (BTHs & BTRs) | 2-Me-BTH, UV-327, UV-328, 5-Cl-BTR, 5-Me-1-H-BTR |
| **G11** | bisphenols (BPs) | BPA, BPAF, BPF, BPS |
| **G12** | butyrate and citrate esters (BEs & CEs) | ATBC, BTHC, TBC, TEC, TXIB, TPIB |
| **G13** | synthetic antioxidants (SAOs) | BHT, BHT-CHO, BHT-COOH, BHT-OH, OC, OMC, Ethanox 703 |

^a^The full names of chemicals are summarized in **Table S3**.

**Table S7.** Distributions of cardiovascular biomarker measurements in participant serum from the screening set.

| **Variables** | **Mean** | **SD** | **Min** | **Percentile** | | | **Max** |
| --- | --- | --- | --- | --- | --- | --- | --- |
|  |  |  |  | **25%** | **50%** | **75%** |  |
| A2M (μg/ml) | 1426 | 601.3 | 548.8 | 1092 | 1305 | 1621 | 5694 |
| Adipsin (μg/ml) | 22.10 | 138.6 | 3.81 | 5.38 | 7.91 | 10.70 | 1611 |
| CRP (μg/ml) | 6.78 | 14.03 | 0.19 | 1.16 | 3.11 | 6.33 | 117.1 |
| Fetuin A (μg/ml) | 426.4 | 297.4 | 221.0 | 297.7 | 359.8 | 451.6 | 3313 |
| Fibrinogen (μg/ml) | 1776 | 695.7 | 840.9 | 1323 | 1702 | 2181 | 5504 |
| Haptoglobin (μg/ml) | 1969 | 3058 | 3.13 | 652.7 | 10518 | 1768 | 21135 |
| L-selectin (μg/ml) | 1.09 | 3.71 | 0.40 | 0.60 | 0.73 | 0.88 | 43.64 |
| PF4 (μg/ml) | 1.61 | 11.59 | 0.07 | 0.14 | 0.25 | 0.45 | 132.6 |
| SAP (μg/ml) | 9.15 | 5.06 | 2.97 | 6.35 | 8.50 | 10.68 | 53.03 |
| vWF (μg/ml) | 59.27 | 159.0 | 7.40 | 16.27 | 33.33 | 52.90 | 1806 |

**Note**: A2M, α2-macroglobulin; CRP, C-reactive protein; PF4, platelet factor 4; SAP, serum amyloid P; vWF, von Willebrand factor.
